# The Missing Enzymes: A Call to Update Pharmacological Profiling Practices for Better Drug Safety Assessment

**DOI:** 10.1021/acs.jmedchem.4c02228

**Published:** 2025-04-02

**Authors:** Monika Maciag, Vardan T. Karamyan

**Affiliations:** Department of Foundational Medical Studies, William Beaumont School of Medicine, Oakland University, Rochester, Michigan 48309, United States

## Abstract

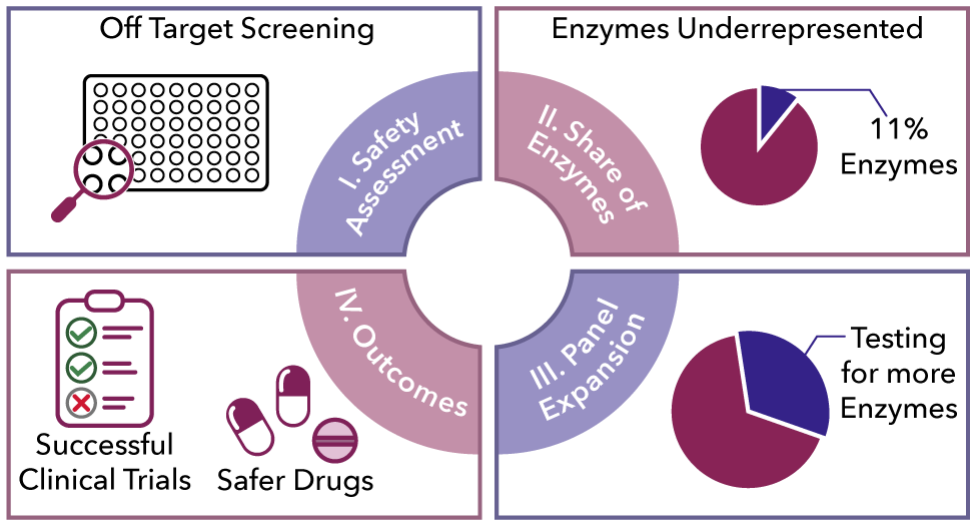

Pharmacological profiling is critical for the development of safe drugs. With increasing awareness of its significance and attempts to share best practices, here we aimed to understand how pharmacological profiling is implemented and reported in the primary literature by analyzing the representation of nonkinase enzymes in selectivity screens. This aspect has been overlooked in previous publications, despite enzymes constituting a significant portion of the pharmacological targets for currently marketed drugs. Our analysis shows that while industry recommendations for improved pharmacological profiling have been widely adopted, enzymes remain largely underrepresented: about a quarter of studies did not include enzymes, and on average, enzymes comprise only 11% of all targets in pharmacological screens. We discuss possible reasons for this shortcoming and provide examples of critical enzymes missing from current screens. We conclude with the notion that selectivity screens should be expanded to include more enzymes to improve drug development and safety.

## Significance

- This perspective provides the first comprehensive analysis of pharmacological profiling practices, with a focus on nonkinase enzymes.
- The underrepresentation of nonkinase enzymes in pharmacological profiling panels is a critical issue for improving drug development.
- The paper calls for the expansion of the current safety panels to include a broader range of enzymes and suggests several candidates associated with adverse effects and high hit rates.
- The potential of artificial intelligence in facilitating pharmacological profiling is discussed.

## Introduction

Drug development remains a long and high-risk process with the majority of candidates failing in the first phase of clinical trials.^1,2^ The primary reasons for failures are a lack of efficacy, affecting nearly half of potential drug candidates, and toxicity observed in 30% of cases.^2−4^ The toxicity of a new drug candidate is typically caused by its primary activity, chemically induced toxicity, or off-target (secondary) effects.^2^ While the first two can be challenging to eliminate, *in vitro* pharmacological profiling during preclinical research offers a practical way to identify off-target activity, predict potential clinical adverse effects, and mitigate associated toxicity to minimize attrition at later stages of drug development.^5^

Pharmacologically, selectivity is defined as the ability of a drug to preferentially affect a particular target over others.^6,7^ In practice, the stronger the preference of a drug to bind or interact with its intended target over others, the more selective it is considered to be. However, designing highly selective compounds is challenging due to the structural and functional similarities among many biological targets.^8,9^ Consequently, most currently used drugs modulate multiple target proteins, resulting in either therapeutic benefits or unwanted adverse effects.^10^ While unintended target interactions can occur with both small-molecule drugs and biologics, these two classes of therapeutics differ significantly. Biologics generally exhibit high specificity and low off-target toxicity, whereas small molecules tend to be more promiscuous in their interactions. Biologics are typically characterized by their high molecular masses and inability to permeate cell membranes. As a result, many pharmacological targets remain inaccessible to biologics.^11^ In contrast, small-molecule drugs, with their smaller size and membrane permeability, can reach intracellular targets and often interact with multiple proteins. On average, small-molecule drugs are estimated to interact with approximately 6 to 12 targets,^5,12,13^ making the challenge of identifying off-targets particularly significant.

To identify nonspecific interactions and optimize the selectivity, it is common practice to screen new investigational small molecules against a broad range of targets, typically during lead generation or optimization. This approach, known as safety pharmacological profiling or secondary pharmacology, mitigates the risk of introducing potentially unsafe drugs into first-in-human studies, and as a result, has become an integral part of the drug development process.^14−16^ Although understanding the pharmacological profile of small molecule drug candidates is critical for early safety assessment and has a significant impact on clinical adverse events, there are no standardized guidelines for selectivity evaluation, leading to considerable variability in practices among drug developers. This topic has been the subject of recent publications from leading pharmaceutical companies^14,17,18^ and regulatory agencies;^19−21^ however, certain aspects of pharmacological profiling and reporting for small molecules have not been adequately explored.

Therefore, the purpose of this perspective article is to examine how pharmacological profiling for new investigational small molecules is reported in the primary literature, which often serves as the initial public forum for reporting new discoveries and discussing critical topics in the field. Additionally, we have extended our analysis to assess and deliberate the representation of nonkinase enzymes in selectivity screens, because this aspect has been largely overlooked in previous publications, despite enzymes constituting a significant portion of pharmacological targets for currently marketed drugs.

## Enzymes – Key Targets in Drug Discovery and Development

**Figure 1:**
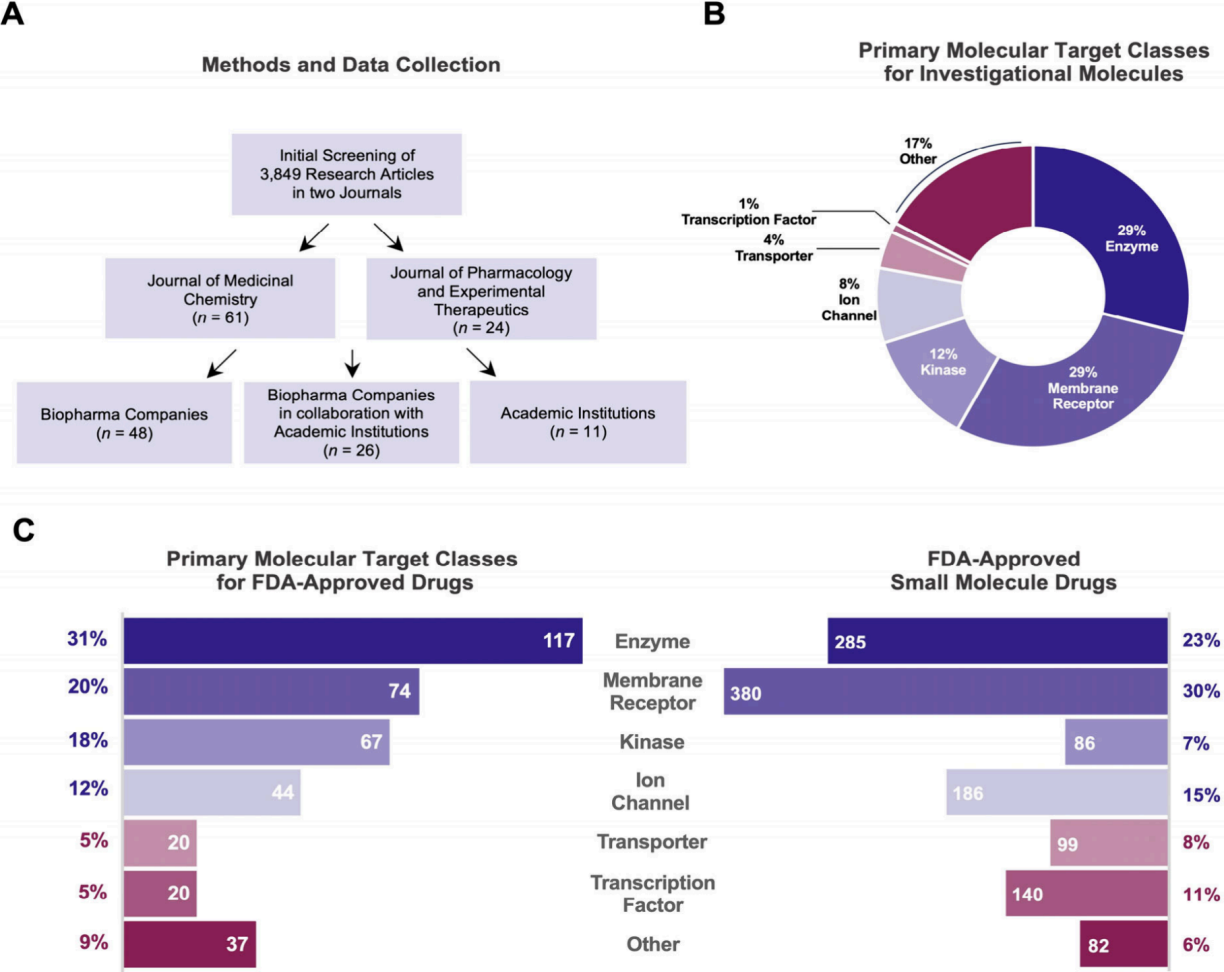
Enzymes are the main targets for the majority of the investigational small molecules and FDA-approved small molecule drugs. (A) Simplified schematic of inclusion criteria for selecting studies from two leading journals. A total of 3,849 articles from the *Journal of Medicinal Chemistry(JMC)* and the *Journal of Pharmacology and Experimental Therapeutics(JPET)* were screened. The primary inclusion criteria focused on publications from biopharma companies or collaborations between companies and academic institutions. Academic articles were considered only if selectivity screening was conducted by an external company (see main text for more details). (B) The share of the primary target class for the investigational molecules reported in the selected 85 articles. Enzymes and membrane receptors are the most common drug targets for the investigational molecules, each accounting for 29% of all targets. (C) The share of primary target class for FDA-approved small molecule drugs. Among 379 primary targets for small molecules, 117 (31%) are enzymes, whereas among 1,258 FDA-approved small molecule drugs, 285 (23%) act through an enzyme. The ChEMBL Database was used for this analysis. A total of 379 distinct, primary pharmacological targets of human origin were identified by screening the database based on the “primary target” of FDA-approved drugs. The data set of FDA-approved drugs included only drugs approved before April 2024, resulting in a total of 1,258 small-molecule drugs.

Recent advances in high-throughput screening and increased awareness of the need for comprehensive pharmacological profiling have led to the routine testing of new drug candidates against a wide range of targets (e.g., receptors, enzymes, ion channels, and transporters). However, due to the lack of clear regulatory requirements and standardization regarding secondary pharmacology, there is a large variability in the representation of targets in selectivity screens among different pharmaceutical companies, and the selection of targets is not often well-justified.^21^ Therefore, to assess the effectiveness of current selectivity profiling practices, we analyzed and manually extracted data from 3,849 peer-reviewed research articles, focusing on investigational small molecule drug candidates, from two leading journals: the *Journal of Medicinal Chemistry* (JMC, published between 2021 and 2023) and the *Journal of Pharmacology and Experimental Therapeutics* (JPET, published between 2019 and 2023), which publish the majority of studies related to the discovery and preclinical evaluation of early clinical drug candidates. Because the process of drug development differs significantly between academia and pharmaceutical companies,^1^ our analysis focused on articles published by biopharma companies or those resulting from the collaboration between companies and academic institutions. Articles from academic units were included only if the selectivity screening was performed by an external company. Additionally, to ensure a broader range of pharmacological targets and classes, we included articles where selectivity screening was performed for at least 30 targets, which is comparable to the size of the core panels used by pharmaceutical companies for primary pharmacological profiling.^18^ This resulted in the selection of 61 articles from *JMC* and 24 articles from *JPET* (Figure 1A, for the list of these articles, see Supporting Information S1).

We began our analysis by identifying the main pharmacological target for the investigational molecules studied in the selected 85 articles, and it was revealed that enzymes and membrane receptors were the most common primary molecular target class, each accounting for 29% of all targets (Figure 1B, Table S2). They were followed by kinases and ion channels accounting for 12% and 8% of all targets, respectively. A similar analysis was carried out for the Food and Drug Administration (FDA)-approved drugs using the ChEMBL Database.^22^ Our focus was on small-molecule drugs with pharmacological targets of human origin. The pharmacological targets were classified according to ChEMBL’s categories including ‘enzyme’, ‘membrane receptor’, ‘ion channel’, ‘transporter’, ‘transcription factor’, and ‘other’ (constitutes epigenetic regulators, secreted proteins, cytosolic proteins, and others). Although the ChEBML Database classifies classic enzymes and kinases together, we have manually analyzed and separated them into two distinct groups. Our analysis revealed that enzymes are the largest pharmacological target class for the current FDA-approved drugs, making up about one-third of all targets (Figure 1C, Tables S3 and S4). Membrane receptors and kinases also ranked highly, each comprising around 20% of all targets. These results are similar to previous reports indicating that enzymes are the most common targets for both FDA-approved drugs and small molecule drug candidates in phase I–III trials in 2023.^18^ Given this and our long-standing interest in enzymes and their modulation for therapeutic purposes,^23−30^ we decided to focus our subsequent analysis on understanding the representation of enzymes in selectivity screens.

## Representation of Enzymes in Pharmacological Profiling in Early Drug Discovery

**Figure 2:**
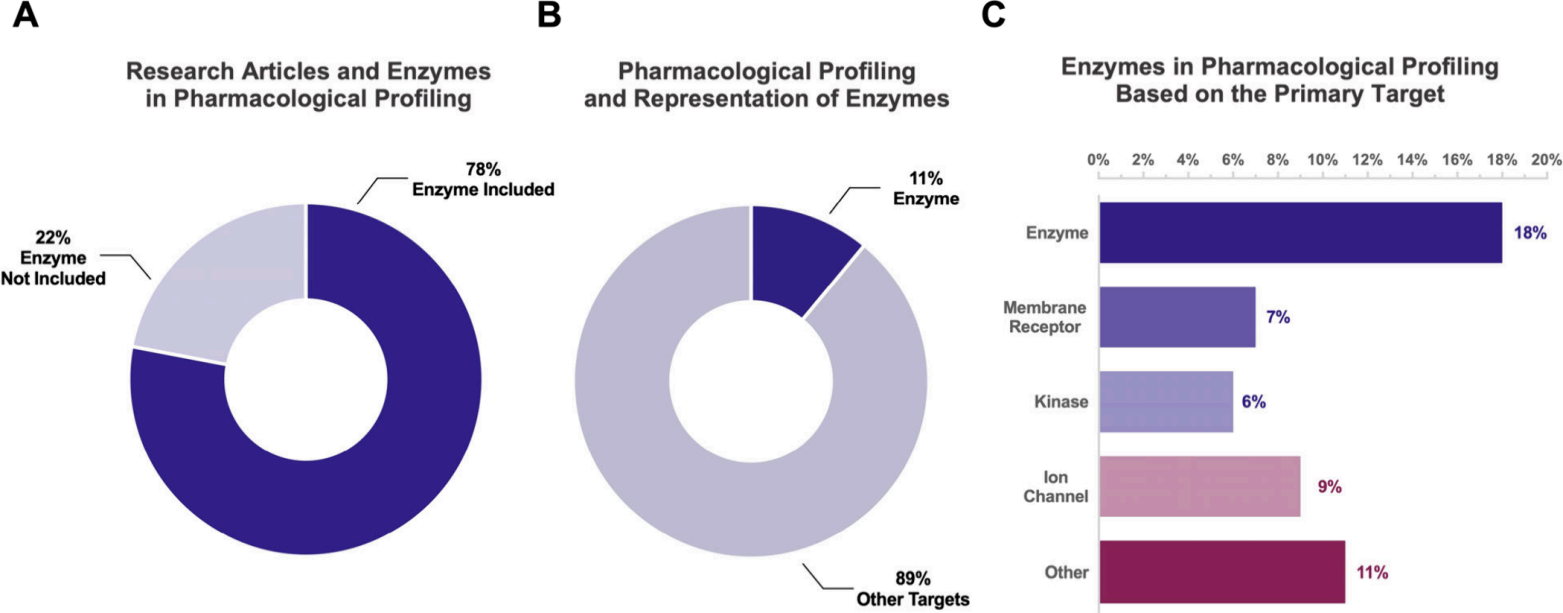
Representation of nonkinase enzymes in pharmacological selectivity studies. (A) The percentage of research articles that included at least one enzyme in the pharmacological profiling of new investigational drug molecules. Of the 85 studies, 19 (22%) did not include any enzyme in the selectivity screen, whereas 66 studies (78%) included at least one enzyme. (B) The share of enzymes compared to all other pharmacological targets in the selectivity screen. Enzymes accounted for 11% of all molecular targets in the selectivity screens of the published studies. (C) When a drug was designed to target an enzyme, the proportion of enzymes in the selectivity screens reached 18%.

Our analysis of the 85 shortlisted articles published in JMC and JPET revealed that approximately one-quarter of the studies did not include any enzymes in the selectivity profiling of the investigational small molecules (Figure 2A, Table S2). Furthermore, in the selectivity profiling screens, enzymes comprised only 11% of all targets (Figure 2B, Table S2). Given that enzymes comprise the largest target class for the investigational small molecules as well as for the currently approved drugs (Figure 1B,C), it is surprising that the share of enzymes in selectivity profiling screens was either absent or so low. This incongruence is further accentuated by findings of a recent study from Amgen which utilized human genetics and pharmacological data to identify off-targets associated with adverse effects and suggested that enzymes (23% nonkinase enzymes and 10% kinases) are implicated in about one-third of adverse events.^31^ Similarly, a recent FDA study analyzing the secondary pharmacology assays submitted with Investigational New Drug (IND) applications confirms our findings and concludes that enzymes generally are tested less frequently than other molecular targets despite having comparable or higher hit rates in the selectivity screens.^19^

In our subsequent analysis, we revealed that when an investigational small molecule was designed to target an enzyme (i.e., the primary pharmacological target is an enzyme), the proportion of enzymes in the selectivity screen was above average, reaching 18% (Figure 2C, Table S2). Conversely, if an investigational molecule was designed to act on a nonenzyme target, the proportion of enzymes in the selectivity screen was usually below average. This divergence may be expected and likely has to do with the tendency of individual companies and laboratories to focus more on molecular targets that have sequence similarity and/or belong to the same family of proteins as the primary pharmacological target of their interest.^17,21^

## The Nature of Enzyme Targets in Pharmacological Profiling Panels

**Figure 3:**
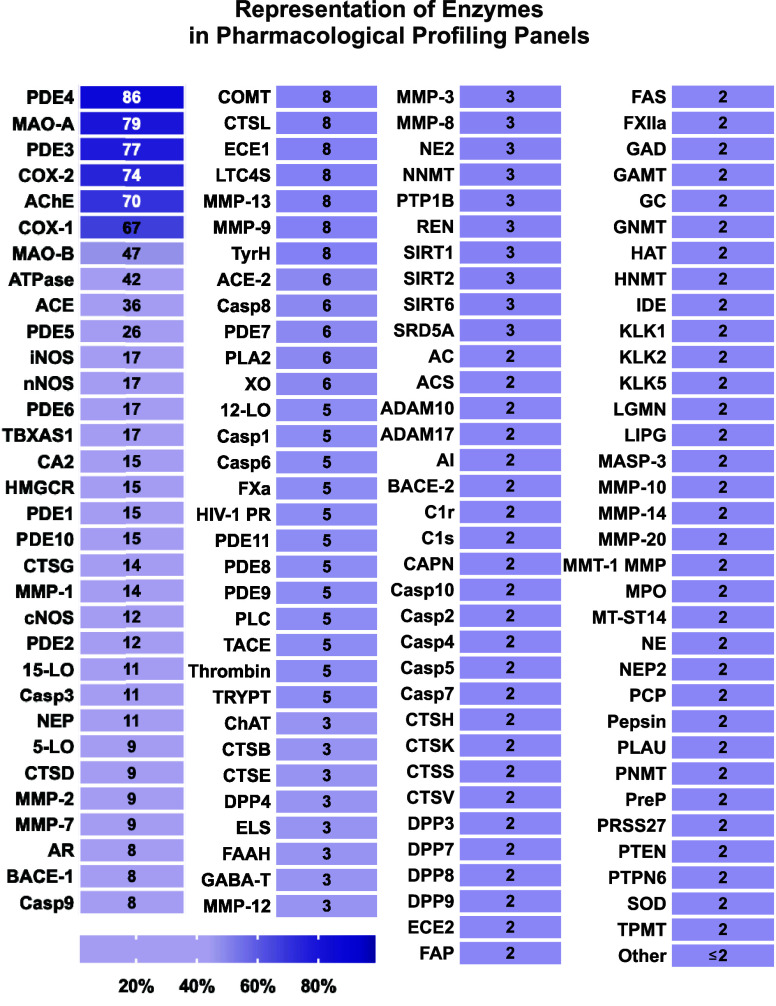
The nature of nonkinase enzyme targets in pharmacological selectivity profiling. Among the 66 studies that included enzymes in their selectivity screens, the most commonly tested enzyme was phosphodiesterase 4 (PDE4), followed by monoamine oxidase A (MAO-A), PDE3, and cyclooxygenase-2 (COX-2). Importantly, nine of the top ten enzymes in this list are collectively recommended by Bowes et al. (2012)^14^ and two subsequent collaborative papers^17,18^ from pharmaceutical companies, suggesting that the guidance has been adopted in the field to some extent. Note that for simplicity, data for different isoforms of PDE were consolidated under one subfamily in this list. Similarly, data for skeletal and heart ATPases were pooled. Abbreviations: **5-LO:** 5-Lipoxygenase, **12-LO:** 12-Lipoxygenase, **15-LO:** 15-Lipoxygenase, **AC:** Adenyl Cyclase, **ACE:** Angiotensin-Converting Enzyme, **ACE-2:** Angiotensin-Converting Enzyme 2, **AChE:** Acetylcholinesterase, **ACS:** Acetyl-CoA Synthetase, **ADAM10:** A Disintegrin and Metalloprotease 10, **ADAM17:** A Disintegrin and Metalloprotease 17, **AI:** Arginase I, **AR:** Aldose Reductase, **ATPase:** ATPase (Skeletal, Heart), **BACE-1:** β-Secretase 1, **BACE-2:** β-Secretase 2, **C1r:** Complement C1r Subcomponent, **C 1s:** Complement C 1s Subcomponent, **CA2:** Carbonic Anhydrase II, **CAPN:** Calpain, **Casp1:** Caspase 1, **Casp2:** Caspase 2, **Casp3:** Caspase 3, **Casp4:** Caspase 4, **Casp5:** Caspase 5, **Casp6:** Caspase 6, **Casp7:** Caspase 7, **Casp8:** Caspase 8, **Casp9:** Caspase 9, **Casp10:** Caspase 10, **ChAT:** Choline Acetyltransferase, **cNOS:** Constitutive Nitric Oxide Synthetase (endothelial), **COMT:** Catechol-O-Methyltransferase, **COX-1:** Cyclooxygenase-1, **COX-2:** Cyclooxygenase-2, **CTSB:** Cathepsin B, **CTSD:** Cathepsin D, **CTSE:** Cathepsin E, **CTSG:** Cathepsin G, **CTSH:** Cathepsin H, **CTSK:** Cathepsin K, **CTSL:** Cathepsin L, **CTSS:** Cathepsin S, **CTSV:** Cathepsin V, **DPP3:** Dipeptidyl Peptidase-3, **DPP4:** Dipeptidyl Peptidase-4, **DPP7:** Dipeptidyl Peptidase-7, **DPP8:** Dipeptidyl Peptidase-8, **DPP9:** Dipeptidyl Peptidase-9, **ECE1:** Endothelin Converting Enzyme 1, **ECE2:** Endothelin Converting Enzyme 2, **ELS:** Elastase, **FAAH:** Fatty Acid Amide Hydrolase, **FAP:** Fibroblast Activation Protein, **FAS:** Fatty Acid Synthase, **FXa:** Factor Xa, **FXIIa:** Factor XIIa, **GABA-T:** GABA Transaminase, **GAD:** Glutamic Acid Decaroboxylase, **GAMT:** Guanidinoacetate *N*-methyltransferase, **GC:** Guanylyl cyclase, **GNMT:** Glycine *N*-methyltransferase, **HAT:** Airway trypsin-like protease, **HIV-1 PR:** HIV-1 Protease, **HMGCR:** 3-Hydroxy-3-Methyl-Glutaryl-Coenzyme A Reductase, **HNMT:** Histamine *N*-Methyltransferase, **IDE:** Insulysin, **iNOS:** Nitric Oxide Synthetase, Inducible, **KLK1:** Kallikrein-1, **KLK2:** Kallikrein-2, **KLK5:** Kallikrein-5, **LGMN:** Legumain, **LIPG:** Endothelial Lipase, **LTC4S:** Leukotriene LTC4 Synthase, **MAO-A:** Monoamine oxidase A, **MAO-B:** Monoamine oxidase B, **MASP-3:** Mannan-binding Lectin-Associated Serine Protease 3, **MMP-1:** Metalloproteinase-1, **MMP-2:** Metalloproteinase-2, **MMP-3:** Metalloproteinase-3, **MMP-7:** Metalloproteinase-7, **MMP-8:** Metalloproteinase-8, **MMP-9:** Metalloproteinase-9, **MMP-10:** Metalloproteinase-10, **MMP-12:** Metalloproteinase-12, **MMP-13:** Metalloproteinase-13, **MMP-14:** Metalloproteinase-14, **MMP-20:** Metalloproteinase-20, **MMT-1 MMP:** Membrane Type 1 Matrix Metalloproteinase, **MPO:** Myeloperoxidase, **MT-ST14:** Matriptase/ST14, **NE:** Neutrophil Elastase, **NE2:** Neutrophil Elastase 2, **NEP:** Metalloproteinase, Neutral Endopeptidase, Neprilysin, **NEP2:** Neprilysin-2, **NNMT:** Nicotinamide *N*-methyltransferase, **nNOS:** Nitric Oxide, Synthase, Neuronal, **PCP:** Pyrrolidone Carboxyl Peptidase, **PDE1:** Phosphodiesterase 1, **PDE2:** Phosphodiesterase 2, **PDE3:** Phosphodiesterase 3, **PDE4:** Phosphodiesterase 4, **PDE5:** Phosphodiesterase 5, **PDE6:** Phosphodiesterase 6, **PDE7:** Phosphodiesterase 7, **PDE8:** Phosphodiesterase 8, **PDE9:** Phosphodiesterase 9, **PDE10:** Phosphodiesterase 10, **PDE11:** Phosphodiesterase 11, **Pepsin:** Pepsin, **PLA2:** Phospholipase A2, **PLAU:** Urokinase, **PLC:** Phospholipase C, **PNMT:** Phenylethanolamine *N*-Methyltransferase, **PreP:** Prolyloligopeptidase, **PRSS27:** Marapsin/Pancreasin, **PTEN:** Phosphatase and Tensin Homologue, **PTP1B:** Protein Tyrosine Phosphatase 1B, **PTPN6:** Tyrosine-Protein Phosphatase Non-Receptor Type 6, **REN:** Renin, **SIRT1:** Sirtuin 1, **SIRT2:** Sirtuin 2, **SIRT6:** Sirtuin 6, **SOD:** Free Radical Scavenger, SOD Mimetic, **SRD5A:** Steroid 5α-reductase, **TACE:** Tumor Necrosis Factor-Alpha Converting Enzyme, **Thrombin:** Thrombin, **TPMT:** Thiopurine *S*-Methyltransferase, **TRYPT:** Tryptase, **TBXAS1:** Thromboxane A Synthase 1, **TyrH:** Tyrosin hydroxylase, **XO:** Xanthine oxidase.

Next, we decided to take a closer look at the specific enzymes that were most frequently screened for the investigational new molecules in the shortlisted studies published in JMC and JPET. Our analysis revealed that the top ten most screened enzymes in the selectivity panels, appearing in 25% to 90% of studies, were phosphodiesterases 3, 4, and 5 (PDE3, PDE4, and PDE5), monoamine oxidases A and B (MAO-A and MAO-B), cyclooxygenases 1 and 2 (COX-1 and COX-2), acetylcholinesterase (AChE), sodium/potassium-adenosine triphosphatase (Na^+^/K^+^-ATPase), and angiotensin-converting enzyme (ACE) (Figure 3, Table S5). Among the 131 enzymes identified in these selectivity screens, only six (PDE3 and PDE4, COX-1 and COX-2, MAO-A, and AChE) were evaluated in more than half of the studies (Figure 3, Table S5).

Currently, the FDA does not specify which targets should be included in an *in vitro* safety profiling panel.^32^ Similarly, the European Medicines Agency (EMA), the counterpart of the FDA in Europe, mentions only the necessity of testing off-targets that are structurally and functionally closely related to the intended target.^33^ The only pharmacological assay mandated by both regulatory authorities is to measure the effects of new compounds on the native ionic current (I_Kr_), or in particular, the human ether-à-go-go-related gene (hERG) potassium channels, which has been linked to fatal *torsade de pointes* arrhythmias.^14,34^ The mandatory inclusion of this assay is a response to the International Conference on Harmonization (ICH), which proposed the S7B guideline requiring hERG sensitivity testing for every new drug.^35,36^ For drugs acting on the central nervous system (CNS), it is also required to include neuronal targets associated with abuse potential, for instance, gamma-aminobutyric acid (GABA) receptors, opioid receptors, and dopamine and serotonin transporters.^37^

The absence of specific regulatory requirements for selectivity profiling has prompted pharmaceutical companies to openly discuss the issue and develop consensus recommendations for off-target pharmacological screening. The first major attempt was a collaboration between AstraZeneca, GlaxoSmithKline, Novartis, and Pfizer, which analyzed the problem and proposed a panel of 44 targets (the so-called Bowes-44 panel) for *in vitro* pharmacological profiling.^14^ This panel includes 24 membrane receptors, eight ion channels, three neurotransmitter transporters, two nuclear hormone receptors, one kinase, and six enzymes that are well-recognized to be associated with adverse effects and toxicity.^14^ The recommended enzymes in the Bowes-44 panel are AChE, COX-1 and COX-2, MAO-A, PDE3A, and PDE4D, which are the top six enzymes that were identified in our analysis and were present in 70% to 90% of the shortlisted studies published in JMC and JPET (Figure 3, Table S5). This finding suggests that recommendations by Bowes and colleagues have been largely adopted in the drug development field, and it is in line with a recent report from a larger coalition of pharmaceutical companies.^18^ Another study published by AbbVie^17^ in 2017 recommended a pharmacological profiling panel that is similar to the Bowes-44 and includes six enzymes that partially overlap with those in the Bowes-44 panel: AChE, ACE, COX-2, MAO-A, PDE3A, and Na^+^/K^+^-ATPase. Notably, ACE and Na^+^/K^+^-ATPase are among the top ten enzymes identified in our analysis; however, they appear in fewer than half of the screens in the shortlisted studies (Figure 3, Table S5). Lastly, MAO-B is listed among the top ten enzymes identified in our analysis but is not included in either the Bowes-44 or AbbVie’s 2017 panels,^14,17^ though it has recently been recommended by the DruSafe leadership group of the International Consortium for Innovation and Quality in Pharmaceutical Development.^18^ In other words, the top ten enzymes identified in our analysis are collectively recommended by a coalition of pharmaceutical companies that have been at the forefront of researching the issue of secondary pharmacology for over a decade. Aside from these top ten enzymes, most other enzymes were tested in only one or a few studies. These findings further confirm that enzymes are not a dominant target class in selectivity screens for new investigational drugs.

It is evident that testing new investigational drugs against all potential targets would not be economically or practically feasible. Typically, the choice of targets in pharmacological profiling screens involves scientific and economic considerations and is guided by their relevance to the disease being studied and/or by clinical evidence linking the targets to adverse events.^14,17^ More specific criteria include (1) tissue distribution, the physiological function of targets, and their potential involvement in adverse side effects (including clinical safety liability); (2) structural or functional similarity of unintended targets to the primary target (e.g., members of the same protein family); (3) availability of an assay and functional conservation of targets across animal species and humans; (4) expected prevalence (hit rate) of off-target activity in the assay; and (5) budget limitations.^5,14,16,17,33^ Given this, it is still surprising that some important enzymes were either vastly under-represented or completely missing from the selectivity screens of the studies that we analyzed. For example, in only one-third of the studies, the new investigational molecules were screened against ACE, despite its well-documented relevance to cardiovascular disorders and the risk of angioedema.^38^ The same could be true for neprilysin (NEP), which was included in about 10% of the screens that we studied but is not a part of the Bowes-44 or similar panels recommended by pharmaceutical companies. NEP is well recognized for its role in cardiovascular function and the risk of angioedema, with some evidence of involvement in the development of Alzheimer’s disease and associated dementias.^38,39^ A similar argument could be made for other peptidases like ACE2, endothelin converting enzyme, dipeptidyl-peptidase 4, neurolysin, prolyl oligopeptidase, and several cathepsins and caspases, which have important physiological and pathophysiological functions and are targets for either approved or in-development drugs. Several of these peptidases were included in the selectivity screens of one or two studies that we analyzed; however, they are not a part of the Bowes-44 or similar panels.

Among other enzymes, carbonic anhydrase 5 (CA5),^40^ 3-hydroxy-3-methylglutaryl-coenzyme A reductase (HMGCR),^41^ thromboxane A synthase 1 (TBXAS1),^31^ HIV-1 protease (HIV-1 PR),^42^ matrix metalloproteinase 9 (MMP-9),^42^ xanthine oxidase (XO),^42^ and quinone reductase 2 (NQO2)^20^ are good examples of important targets for selectivity panels because of their association with a high risk of adverse events or a high hit rate. Despite their significance, they were identified in only one or a few studies we analyzed (Figure 3, Table S5).

## CROs Shape Pharmacological Profiling Practices

**Figure 4:**
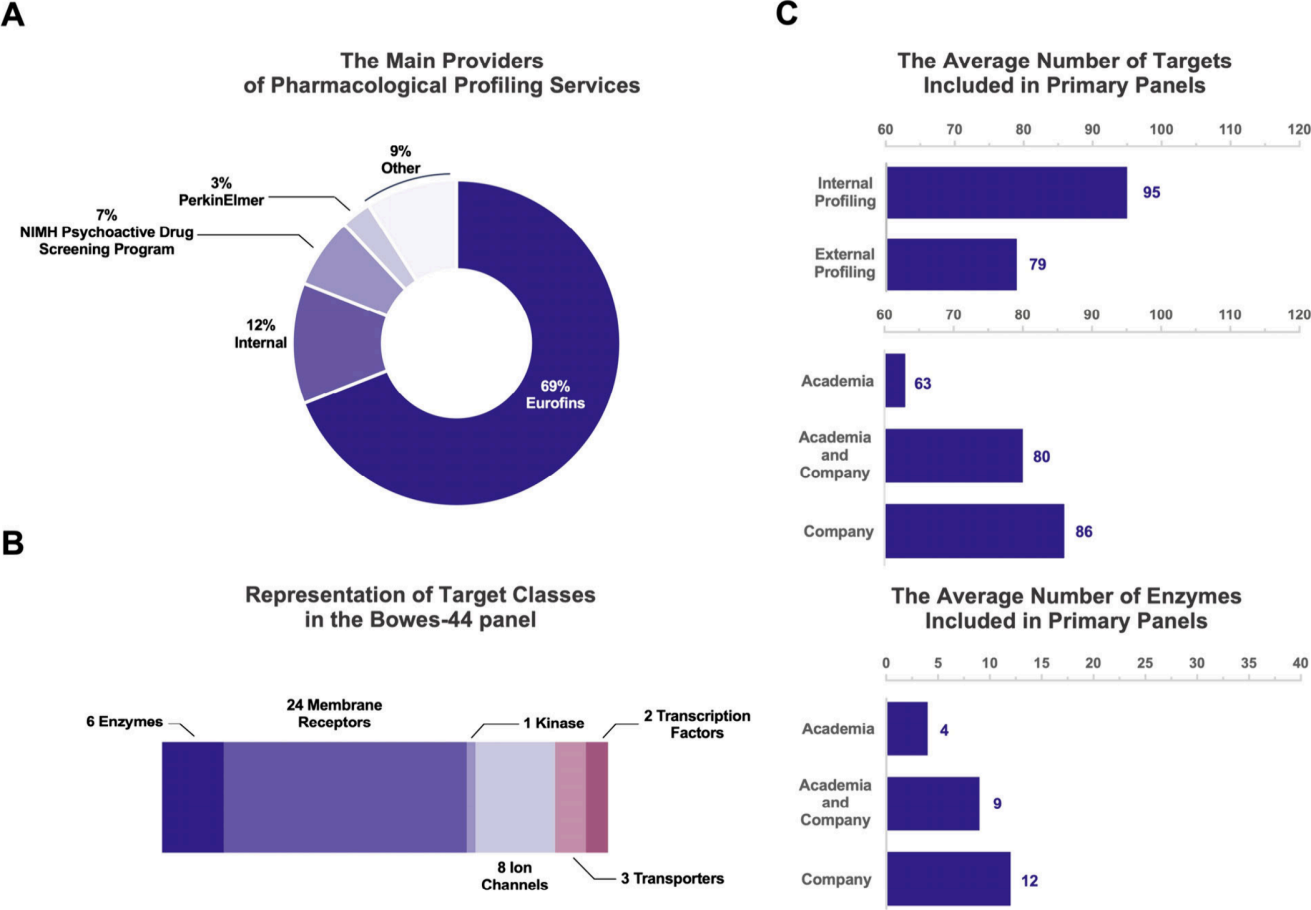
Pharmacological profiling screens, the main providers and extent of use. (A) The analysis of shortlisted 85 studies published in JMC and JPET indicates that only in 12% of the studies selectivity screening was carried out internally. The majority of studies outsourced the screening to external companies, with Eurofins being the dominant provider of such services. (B) The top six enzymes identified in our analysis are also featured in predefined pharmacological profiling panels, such as Eurofins’ SafetyScreen44, which mirrors the Bowes-44 panel.^14^ (C) Our analysis indicates that the total number of targets in pharmacological profiling screens varies between studies originating from academia vs. pharmaceutical companies. The difference also translates to the number of enzymes included in the selectivity screen.

Interestingly, our analysis of the shortlisted studies published in JMC and JPET revealed that close to 70% of pharmacological profiling screening was carried out by contract research organization (CRO) Eurofins,^43^ while only one-tenth was done internally (Figure 4A, Table S2). The remaining screens were performed by other companies or external programs such as PerkinElmer and the NIH Psychoactive Drug Screening Program. The dominance of one or few sources for pharmacological profiling and/or the widespread adoption of the Bowes-44 panel may explain why the top six enzymes ranked in our analysis are also included in such predefined panels for pharmacological profiling. For example, Eurofins’ standardized panel called SafetyScreen44, is identical to the Bowes-44 panel (Figure 4B).^14^ Access to these companies and panels offers clear advantages for drug developers; however, it may also carry the risk of excluding important targets. A good alternative to overcoming this issue could be a tiered approach to selectivity profiling that starts from a core primary panel of a limited number of targets, followed by one or more larger secondary panels. The primary panel, typically smaller and composed of diverse targets with strong historical links to clinical outcomes and high hit rates, serves to evaluate compounds for the promiscuity of interaction with multiple targets. This stage is important not only for safety profiling but also for lead optimization, guiding medicinal chemistry efforts, and elucidating structure–activity relationships.^18^ Subsequently, compounds are tested against broader secondary panels encompassing diverse targets that are clearly associated with potential adverse clinical outcomes, thereby enabling the identification of safety concerns.^18^ The selection of targets for both early screening panels and broader secondary pharmacology screens can vary significantly between organizations. It is shaped by several factors, including institutional experience, the hit rates of current and previous targets, and the primary target class (e.g., screening against closely related family members).^5,17^ Other considerations include organ prioritization—vital organs or systems essential for sustaining life, such as the cardiovascular, respiratory, or CNS—and the therapeutic area.^5,44^ It should be noted that this tiered approach was not used in the studies that we analyzed (no explicit description or mention of it); however, it is a tactic that has been recommended and practiced by some pharmaceutical companies.^18,42,45,46^ Notably, our analysis revealed that when pharmacological profiling was conducted at least partially in-house, the panel included more targets—around 95 on average—compared to those that were run by external organizations, which averaged 79 targets (Figure 4C, Table S2). Another interesting observation in our analysis is that the studies originating from pharmaceutical companies utilized selectivity screens ranging from 30 to over 250 targets with an average of around 80 targets. This highlights the substantial variation in the panel size between different companies. In contrast, in studies originating from academic institutions, where screening was outsourced to a CRO, the range spanned from 40 to 85, with an average of around 60 targets (Figure 4C, Table S2). This average number of total targets for selectivity profiling aligns well with recent reports from pharmaceutical companies^18^ and the FDA.^19^ The noted difference in the total number of targets between pharmaceutical companies and academia also translates into the representation of enzymes—selectivity screens used by pharmaceutical companies had on average 12 enzymes, whereas academic institutions had 4 (Figure 4C, Table S2). One reason for these observations could be that most studies originating from academic institutions report on molecules in the early stages of lead optimization, whereas pharmaceutical companies report on more advanced lead compounds, hence providing more detailed information about pharmacological selectivity. Additionally, these differences may be influenced by the nature of the primary pharmacological targets and budgetary limitations.

## Concluding Remarks

Pharmacological profiling or secondary pharmacology is crucial for identifying potential hazards and reducing clinical risks during drug development. Over the past two decades, efforts to enhance off-target screening strategies have led to significant improvements, with recently marketed small-molecule drugs showing significantly fewer off-target interactions compared to those approved over a decade ago.^18^ Although the literature is continuously updated with new information on high-risk adverse effect targets and emerging guidelines, our work specifically focuses on nonkinase enzymes, which despite being the largest target class for FDA-approved drugs, are often underrepresented in pharmacological profiling screens. Our finding is further highlighted by recent estimates showing that enzymes remain the leading target class for new investigational molecules in phase I to III trials carried out by the largest pharmaceutical companies.^18^ The primary reason for the low representation of enzymes, as well as the consistent testing of the same six enzymes across the studies that we analyzed, is likely a consequence of the widespread adoption of the Bowes-44 panel^14^ by pharmaceutical companies and CROs. Undoubtedly, the Bowes-44 panel was a milestone, marking the first systematic effort to identify the most critical targets for selective screening in early drug development. However, recent research suggests that other enzymes beyond those included in the Bowes-44 panel are equally, if not more, strongly associated with high-risk adverse effects.^20,31,40,42^ The latest industry-wide analysis of pharmacological profiling by the DruSafe leadership group,^18^ has expanded the enzyme portfolio beyond the Bowes-44 panel by including three additional enzymes: MAO-B, cathepsin D (CTSD), and MALT1 paracaspase (MALT1). However, the representation of enzymes still remains low, accounting for only 12% of all targets in this most recently recommended panel.

A positive observation from our analysis is that investigational molecules designed to interact with enzymes were tested against a larger number of enzyme targets compared to the molecules targeting nonenzymes. This supports the general principle that investigational drugs should be evaluated against targets that are similar to their primary pharmacological target.^17^ The use of standardized pharmacological profiling panels ensures a consistent approach across the drug development industry. This is reflected in our analysis, where the top ten screened enzymes align perfectly with the recommendations of working groups from the leading pharmaceutical companies.^14,17,18^ However, this uniform approach may also have some negative outcomes, such as repetitive testing of the same targets across different studies, which may sometimes lead to overlooking drug-specific factors. Consequently, critical off-target effects could be missed, and a problem might be further exacerbated by the reliance on CROs. Therefore, individual laboratories may benefit from modifying target selection based on their specific needs and prior experience.

CROs offer efficient pharmacological profiling, which explains why most investigational molecules were tested through these external programs. Our findings are consistent with a survey of 18 companies, which revealed that most outsource pharmacological profiling to CROs, while only about one-fourth conduct the screening internally.^18^ Interestingly, when pharmacological profiling was conducted at least partially in-house, the panel tended to include a broader range of targets compared to those run by external organizations. This likely reflects the advantage of in-house screening, where comprehensive target selection and specialized expertise are more readily available, in contrast to the more generalized approach of CRO-run screenings. Additionally, we observed that academic institutions, even when outsourcing selectivity profiling to CROs, typically used narrower panels with fewer enzyme targets compared with pharmaceutical companies. This trend is likely driven by budget constraints inherent to academic research.

A great example of progress in secondary pharmacological profiling may be the case of kinase selectivity, where the number of kinases represented in panels has grown significantly from none^17^ or just one kinase^14^ to 20 in the most recent recommended panel.^18^ The human genome encodes over 500 kinases,^47^ and because of the structural similarity of the kinase active sites,^48^ it is well-recognized that developing molecules that specifically target an individual kinase without affecting others remains a significant challenge.^48^ The example of kinases also shows that selectivity profiling extends beyond safety assessment, as it is essential for understanding the overlap between off-target effects and multitarget activity.^49,50^ To better understand these relationships, pharmaceutical companies and CROs have heavily invested in kinase profiling programs,^51^ leading to the development of high-throughput and high-content screening methods covering the entire kinome. For example, investigators at AbbVie have recommended a panel of 95 kinases,^52^ incorporating safety-related data from the literature and institutional expertise built over years of discovery efforts. This panel complements their secondary pharmacology panel^17^ and is designed to evaluate not only kinase-targeting drugs but also compounds targeting other classes of molecules. Similarly, the Reaction Biology Kinase Selectivity Profiling Panel^53^ provides access to a comprehensive collection of over 700 kinases. This broad screening approach was also revealed in our analysis, showing that kinase-targeting molecules were tested, on average, against over 220 kinases (Table S6) and highlights a significant difference in the tactic to selectivity assessment for kinase-targeting compared to other drug candidates.

Given the high-cost demands of obtaining experimental results on such a scale, recent efforts have shifted toward using artificial intelligence and machine learning techniques to predict drug toxicity. One notable initiative is the PanScreen tool,^54^ which integrates deep learning with traditional structure-based modeling techniques. Another example is the work by Ietswaart and colleagues from Novartis,^55^ who have employed machine learning to develop models predicting adverse effects based on *in vitro* pharmacological profiles. While computational models offer a potential alternative to traditional *in vitro* selectivity screening, it is important to recognize a significant limitation of these methods: machine learning models rely on the data on which they are trained on. If a specific molecule–target–adverse effect combination has not been previously encountered in the training data, the model may fail to identify it. Furthermore, it is relatively easy to predict interactions between small molecules and orthosteric binding sites of pharmacological targets because there is detailed structural information for most targets. However, this is not the case when it comes to allosteric binding sites, because most of them are unidentified or poorly understood.^5,56^ One potential solution for generating large-scale data is fostering cooperation among federal agencies, academic institutions, and biopharmaceutical companies. By combining their expertise, resources, and data, these collaborations can create robust and diverse data sets, providing a strong platform for training artificial intelligence and machine learning models. One such initiative is the *Tox21* program, a collaborative effort established through a federal partnership between the U.S. agencies and the FDA.^57,58^ The *Tox21* program has led to the development of *in vitro* assays for quantitative high-throughput screening, resulting in a comprehensive toxicity database based on data from the evaluation of over 10,000 compounds. The extensive *Tox21* library has been then utilized to develop several machine learning models, including those created as part of the *Tox21*,^59,60^ and newer models^61,62^ to predict the toxicological profiles of novel chemicals.

In summary, the primary aim of our study was to present and advocate for the revision of current pharmacological selectivity panels used to assess off-target effects. While this topic has been extensively discussed in recent publications, our study is the first to specifically focus on nonkinase enzymes and their representation in pharmacological selectivity screens. Furthermore, the collected data rely on a comprehensive evaluation of published literature, rather than internal data from pharmaceutical companies, demonstrating that publicly accessible, published data are reported as robust and reliable as the proprietary data sets typically collected by companies. Based on our analysis it is evident that nonkinase enzymes as a target class are underrepresented in most selectivity screens. We believe it is crucial to expand the range of enzyme targets in safety panels and to update standardized panels with the latest data on the association between enzymes and the high risk of adverse effects. A good starting point could be the group of enzymes summarized in Table 1, which have been reported in the literature for association with adverse effects or high hit rates and are among the top ∼20% of most screened enzymes revealed in our analysis.

**Table 1:** A List of Enzymes Proposed for Consideration to Include in Safety Pharmacological Panels, Selected Based on Their Association with Safety Liabilities or a High Hit Rate

target	adverse effects associated with inhibition or deficiency	representation in the screened panel (refer to Figure 3)	hit rate (%) as reported by Brennan et al. (2024)^18^	hit rate (%) as reported by FDA (2022)^19^	refs
5-Lipoxygenase (5-LO)^a^	Immunomodulation	9%	14%	20%	(63, 64)
	Deficiency may contribute to bladder cancer progression				
Carbonic Anhydrase II (CA2)	Aplastic anemia	15%	1%	2%	(40, 65)
	Deficiency contributes to osteopetrosis, renal tubular acidosis, and brain calcification				
3-Hydroxy-3-Methylglutaryl-Coenzyme A Reductase (HMGCR)	Myalgia	15%	1%	0%	(41, 66−68)
	Increased risk of type 2 diabetes				
	Neurocognitive adverse effects				
Matrix Metalloproteinase 1 (MMP-1)^b^	Musculoskeletal toxic effects	14%	4%	3%	(69, 70)
Phosphodiesterase 5 (PDE5)^c^	Cardiovascular risk	26%	12%	10%	(71−73)
	Headache				
	Flushing				
Phosphodiesterase 10 (PDE10)	Somnolence	15%	17%	6%	(74, 75)
	Dystonia				
Thromboxane A Synthase 1 (TBXAS1)	Platelet aggregation	17%	16%	2%	(31, 76−78)
	Deficiency contributes to Ghosal hematodiaphyseal dysplasia syndrome				

a 12-Lipoxygenase (12-LO) and 15-lipoxygenase (15-LO) may also be considered due to their high representation in our analysis and a significant hit rate reported by Brennan et al.^18^

b Matrix metalloproteinase 9 (MMP-9) may also be considered due to the high hit rate.^42^

c Phosphodiesterase 1 (PDE1), phosphodiesterase 2 (PDE2), and phosphodiesterase 6 (PDE6) may also be considered due to their high representation in our analysis and a significant hit rate reported by Brennan et al.^18^ and FDA.^19^

Importantly, all of the suggested enzymes are recombinantly produced and commercially available, and have established assays for measuring enzymatic activity that can be modified for high-throughput screening. It should also be noted that current analytical techniques, e.g., modern mass spectrometry methodologies, make it possible to evaluate the activity of enzymes for which no fluorogenic or labeled substrates are available and were traditionally difficult to study.

We recognize that the adoption of this idea and inclusion of a broader range of enzymes in selectivity screens will require discussions of the under-representation of enzymes among a wider group of stakeholders in the drug development field, and we hope that our paper will serve as a catalyst to initiate these conversations.

Ideally, the equally rigorous practices and comprehensive panel approaches currently applied to testing kinase inhibitors could be extended to other drug classes, with an expectation of achieving better safety and lower attrition of drug candidates at later stages of drug development. While this may extend the drug development timeline and increase the cost in the short term, it could significantly enhance the predictability of clinical outcomes and have a substantially positive impact in the long run. With the accumulation of safety, genetics, structural, and associated knowledge, molecular modeling and artificial-intelligence-based methods could offer quicker and cheaper ways to conduct pharmacological profiling for some of the most common targets.

## Supporting Information Available

Supporting Information S1: the list of articles, published in JMC and JPET, used for this study (PDF)
Tables S2, S5, and S6: pharmacological profiling data, from articles published in JMC and JPET, used for this study. Tables S3 and S4: data from the ChEMBL Database related to the primary targets for FDA-approved drugs (XLSX)

## Abbreviations Used

ACE: angiotensin-converting enzyme
AChE: acetylcholinesterase
CA5: carbonic anhydrase 5
CNS: central nervous system
COX-1: cyclooxygenase 1
COX-2: cyclooxygenase 2
CRO: contract research organization
CTSD: cathepsin D
EMA: European Medicines Agency
FDA: Food and Drug Administration
GABA: Gamma-aminobutyric acid
hERG: human ether-à-go-go-related gene
HIV-1 PR: HIV-1 protease
HMGCR: 3-hydroxy-3-methylglutaryl–coenzyme A reductase
ICH: International Conference on Harmonization
I_Kr_: Native ionic current potassium channel
IND: Investigational New Drug
*JMC*: *Journal of Medicinal Chemistry*
*JPET*: *Journal of Pharmacology and Experimental Therapeutics*
MALT1: MALT1 paracaspase
MAO-A: monoamine oxidase A
MAO-B: monoamine oxidase B
MMP-9: matrix metalloproteinase 9
Na^+^/K^+^-ATPase: sodium/potassium-adenosine triphosphatase
NEP: neprilysin
NQO2: quinone reductase 2
PDE3: phosphodiesterases 3
PDE4: phosphodiesterases 4
PDE5: phosphodiesterases 5
TBXAS1: thromboxane A synthase 1
XO: xanthine oxidase

## References

[ref1] Takebe T.; Imai R.; Ono S. The Current Status of Drug Discovery and Development as Originated in United States Academia: The Influence of Industrial and Academic Collaboration on Drug Discovery and Development. Clin Transl Sci. 2018, 11 (6), 597–606. 10.1111/cts.12577. 29940695 PMC6226120

[ref2] Sun D.; Gao W.; Hu H.; Zhou S. Why 90% of Clinical Drug Development Fails and How to Improve It?. Acta Pharm. Sin B 2022, 12 (7), 3049–3062. 10.1016/j.apsb.2022.02.002. 35865092 PMC9293739

[ref3] Dowden H.; Munro J. Trends in Clinical Success Rates and Therapeutic Focus. Nat. Rev. Drug Discov 2019, 18 (7), 495–496. 10.1038/d41573-019-00074-z. 31267067

[ref4] Harrison R. K. Phase II and Phase III Failures: 2013–2015. Nat. Rev. Drug Discov 2016, 15 (12), 817–818. 10.1038/nrd.2016.184. 27811931

[ref5] Van Vleet T. R.; Liguori M. J.; Lynch J. J. III; Rao M.; Warder S. Screening Strategies and Methods for Better Off-Target Liability Prediction and Identification of Small-Molecule Pharmaceuticals. SLAS Discovery 2019, 24 (1), 1–24. 10.1177/2472555218799713. 30196745

[ref6] Mencher S. K.; Wang L. G. Promiscuous Drugs Compared to Selective Drugs (Promiscuity Can Be a Virtue). BMC Clin Pharmacol 2005, 5, 3 10.1186/1472-6904-5-3. 15854222 PMC1090568

[ref7] Kawasaki Y.; Freire E. Finding a Better Path to Drug Selectivity. Drug Discov Today 2011, 16 (21–22), 985–990. 10.1016/j.drudis.2011.07.010. 21839183 PMC3210374

[ref8] Emmerich C. H.; Gamboa L. M.; Hofmann M. C. J.; Bonin-Andresen M.; Arbach O.; Schendel P.; Gerlach B.; Hempel K.; Bespalov A.; Dirnagl U.; Parnham M. J. Improving Target Assessment in Biomedical Research: The GOT-IT Recommendations. Nat. Rev. Drug Discov 2021, 20 (1), 64–81. 10.1038/s41573-020-0087-3. 33199880 PMC7667479

[ref9] Huggins D. J.; Sherman W.; Tidor B. Rational Approaches to Improving Selectivity in Drug Design. J. Med. Chem. 2012, 55 (4), 1424–1444. 10.1021/jm2010332. 22239221 PMC3285144

[ref10] Wang T.; Pulkkinen O. I.; Aittokallio T. Target-Specific Compound Selectivity for Multi-Target Drug Discovery and Repurposing. Front Pharmacol 2022, 13. 10.3389/fphar.2022.1003480 PMC9549418 36225560

[ref11] Oo C.; Kalbag S. S. Leveraging the Attributes of Biologics and Small Molecules, and Releasing the Bottlenecks: A New Wave of Revolution in Drug Development. Expert Rev. Clin Pharmacol 2016, 9 (6), 747–749. 10.1586/17512433.2016.1160778. 26933755

[ref12] Peón A.; Naulaerts S.; Ballester P. J. Predicting the Reliability of Drug-Target Interaction Predictions with Maximum Coverage of Target Space. Sci. Rep 2017, 7 (1), 3820 10.1038/s41598-017-04264-w. 28630414 PMC5476590

[ref13] Mestres J.; Gregori-Puigjané E. Conciliating Binding Efficiency and Polypharmacology. Trends Pharmacol. Sci. 2009, 30 (9), 470–474. 10.1016/j.tips.2009.07.004. 19717193

[ref14] Bowes J.; Brown A. J.; Hamon J.; Jarolimek W.; Sridhar A.; Waldron G.; Whitebread S. Reducing Safety-Related Drug Attrition: The Use of in Vitro Pharmacological Profiling. Nat. Rev. Drug Discov 2012, 11 (12), 909–922. 10.1038/nrd3845. 23197038

[ref15] Sutherland J. J.; Yonchev D.; Fekete A.; Urban L. A Preclinical Secondary Pharmacology Resource Illuminates Target-Adverse Drug Reaction Associations of Marketed Drugs. Nat. Commun. 2023, 14 (1), 4323 10.1038/s41467-023-40064-9. 37468498 PMC10356841

[ref16] Whitebread S.; Dumotier B.; Armstrong D.; Fekete A.; Chen S.; Hartmann A.; Muller P. Y.; Urban L. Secondary Pharmacology: Screening and Interpretation of off-Target Activities - Focus on Translation. Drug Discov Today 2016, 21 (8), 1232–1242. 10.1016/j.drudis.2016.04.021. 27140035

[ref17] Lynch J. J.; Van Vleet T. R.; Mittelstadt S. W.; Blomme E. A. G. Potential Functional and Pathological Side Effects Related to Off-Target Pharmacological Activity. J. Pharmacol Toxicol Methods 2017, 87, 108–126. 10.1016/j.vascn.2017.02.020. 28216264

[ref18] Brennan R. J.; Jenkinson S.; Brown A.; Delaunois A.; Dumotier B.; Pannirselvam M.; Rao M.; Ribeiro L. R.; Schmidt F.; Sibony A.; Timsit Y.; Sales V. T.; Armstrong D.; Lagrutta A.; Mittlestadt S. W.; Naven R.; Peri R.; Roberts S.; Vergis J. M.; Valentin J. P. The State of the Art in Secondary Pharmacology and Its Impact on the Safety of New Medicines. Nat. Rev. Drug Discov 2024, 23 (7), 525–545. 10.1038/s41573-024-00942-3. 38773351

[ref19] Scott C.; Dodson A.; Saulnier M.; Snyder K.; Racz R. Analysis of Secondary Pharmacology Assays Received by the US Food and Drug Administration. J. Pharmacol Toxicol Methods 2022, 117, 107205 10.1016/j.vascn.2022.107205. 35926773

[ref20] Dodson A.; Mi K.; Russo D. P.; Scott C.; Saulnier M.; Snyder K.; Racz R. Aggregation and Analysis of Secondary Pharmacology Data from Investigational New Drug Submissions at the US Food and Drug Administration. J. Pharmacol Toxicol Methods 2021, 111, 107098 10.1016/j.vascn.2021.107098. 34229067

[ref21] Papoian T.; Chiu H. J.; Elayan I.; Jagadeesh G.; Khan I.; Laniyonu A. A.; Li C. X.; Saulnier M.; Simpson N.; Yang B. Secondary Pharmacology Data to Assess Potential Off-Target Activity of New Drugs: A Regulatory Perspective. Nat. Rev. Drug Discov 2015, 14 (4), 294 10.1038/nrd3845-c1. 25792260

[ref22] ChEMBL Database. www.ebi.ac.uk/chembl/ (accessed Sep 15, 2024).

[ref23] Jayaraman S.; Kocot J.; Esfahani S. H.; Wangler N. J.; Uyar A.; Mechref Y.; Trippier P. C.; Abbruscato T. J.; Dickson A.; Aihara H.; Ostrov D. A.; Karamyan V. T. Identification and Characterization of Two Structurally Related Dipeptides That Enhance Catalytic Efficiency of Neurolysin. J. Pharmacol Exp Ther 2021, 379 (2), 191–202. 10.1124/jpet.121.000840. 34389655 PMC8626779

[ref24] Jayaraman S.; Al Shoyaib A.; Kocot J.; Villalba H.; Alamri F. F.; Rashid M.; Wangler N. J.; Chowdhury E. A.; German N.; Arumugam T. V.; Abbruscato T. J.; Karamyan V. T. Peptidase Neurolysin Functions to Preserve the Brain after Ischemic Stroke in Male Mice. J. Neurochem 2020, 153 (1), 120–137. 10.1111/jnc.14864. 31486527 PMC7056597

[ref25] Wangler N. J.; Jayaraman S.; Zhu R.; Mechref Y.; Abbruscato T. J.; Bickel U.; Karamyan V. T. Preparation and Preliminary Characterization of Recombinant Neurolysin for in Vivo Studies. J. Biotechnol. 2016, 234, 105–115. 10.1016/j.jbiotec.2016.07.007. 27496565

[ref26] Karamyan V. T.; Gadepalli R.; Rimoldi J. M.; Speth R. C. Brain AT1 Angiotensin Receptor Subtype Binding: Importance of Peptidase Inhibition for Identification of Angiotensin II as Its Endogenous Ligand. J. Pharmacol Exp Ther 2009, 331 (1), 170–177. 10.1124/jpet.109.157461. 19587313

[ref27] Karamyan V. T.; Speth R. C. Enzymatic Pathways of the Brain Renin-Angiotensin System: Unsolved Problems and Continuing Challenges. Regul. Pept. 2007, 143 (1–3), 15–27. 10.1016/j.regpep.2007.03.006. 17493693 PMC7114358

[ref28] Shi K.; Bagchi S.; Bickel J.; Esfahani S. H.; Yin L.; Cheng T.; Karamyan V. T.; Aihara H. Structural Basis of Divergent Substrate Recognition and Inhibition of Human Neurolysin. Sci. Rep 2024, 14 (1), 8420 10.1038/s41598-024-67639-w. 39117724 PMC11310207

[ref29] Rahman M. S.; Kumari S.; Esfahani S. H.; Nozohouri S.; Jayaraman S.; Kinarivala N.; Kocot J.; Baez A.; Farris D.; Abbruscato T. J.; Karamyan V. T.; Trippier P. C. Discovery of First-in-Class Peptidomimetic Neurolysin Activators Possessing Enhanced Brain Penetration and Stability. J. Med. Chem. 2021, 64 (17), 12705–12722. 10.1021/acs.jmedchem.1c00759. 34436882 PMC9295256

[ref30] Esfahani S. H.; Jayaraman S.; Karamyan V. T. Is Diminazene an Angiotensin-Converting Enzyme 2 (ACE2) Activator? Experimental Evidence and Implications. J. Pharmacol Exp Ther 2022, 383 (2), 149–156. 10.1124/jpet.122.001339. 36507848 PMC9553104

[ref31] Deaton A. M.; Fan F.; Zhang W.; Nguyen P. A.; Ward L. D.; Nioi P. Rationalizing Secondary Pharmacology Screening Using Human Genetic and Pharmacological Evidence. Toxicol. Sci. 2019, 167 (2), 593–603. 10.1093/toxsci/kfy265. 30346593 PMC6358245

[ref32] U.S. Food & Drug Administration. IND Applications for Clinical Investigations: Pharmacology and Toxicology (PT) Information. https://www.fda.gov/drugs/investigational-new-drug-ind-application/ind-applications-clinical-investigations-pharmacology-and-toxicology-pt-information (accessed Sep 15, 2024).

[ref33] Guideline on strategies to identify and mitigate risks for first-in-human and early clinical trials with investigational medicinal products. https://www.ema.europa.eu/en/documents/scientific-guideline/guideline-strategies-identify-and-mitigate-risks-first-human-and-early-clinical-trials-investigational-medicinal-products-revision-1_en.pdf (accessed Sep 15, 2024). 10.1111/bcp.13550 PMC6005602 29451320

[ref34] Hefti F. F. Requirements for a Lead Compound to Become a Clinical Candidate. BMC Neurosci. 2008, 9, (Suppl 3), . 10.1186/1471-2202-9-S3-S7 PMC2604885 19091004

[ref35] U.S. Food & Drug Administration. S7B Nonclinical Evaluation of the Potential for Delayed Ventricular Repolarization (QT Interval Prolongation) by Human Pharmaceuticals. https://www.fda.gov/regulatory-information/search-fda-guidance-documents/s7b-nonclinical-evaluation-potential-delayed-ventricular-repolarization-qt-interval-prolongation (accessed Sep 15, 2024).

[ref36] European Medicines Agency. ICH guideline E14/S7B: clinical and Nonclinical Evaluation of QT/QTc Interval Prolongation and Proarrhythmic Potential - questions and answers. https://www.ema.europa.eu/en/documents/scientific-guideline/ich-guideline-e14s7b-clinical-and-nonclinical-evaluation-qtqtc-interval-prolongation-and-proarrhythmic-potential-questions-and-answers-step-5_en.pdf (accessed Sep 15, 2024).

[ref37] U.S. Food & Drug Administration. Assessment of Abuse Potential of Drugs. https://www.fda.gov/regulatory-information/search-fda-guidance-documents/assessment-abuse-potential-drugs (accessed Sep 15, 2024).

[ref38] Owens R. E.; Oliphant C. S. Angioedema Spotlight: A Closer Examination of Sacubitril/Valsartan Safety Results. J. Am. Board Fam Med. 2017, 30 (4), 556–557. 10.3122/jabfm.2017.04.170111. 28720639

[ref39] Chen C.; Ding L.; Fu F.; Xiao J. Updated Insights on Dementia-Related Risk of Sacubitril/Valsartan: A Real-World Pharmacovigilance Analysis. CNS Neurosci Ther 2023, 29 (9), 2548–2554. 10.1111/cns.14195. 36971193 PMC10401082

[ref40] Smit I. A.; Afzal A. M.; Allen C. H. G.; Svensson F.; Hanser T.; Bender A. Systematic Analysis of Protein Targets Associated with Adverse Events of Drugs from Clinical Trials and Postmarketing Reports. Chem. Res. Toxicol. 2021, 34 (2), 365–384. 10.1021/acs.chemrestox.0c00294. 33351593

[ref41] Duran-Frigola M.; Aloy P. Analysis of Chemical and Biological Features Yields Mechanistic Insights into Drug Side Effects. Chem. Biol. 2013, 20 (4), 594–603. 10.1016/j.chembiol.2013.03.017. 23601648

[ref42] Bendels S.; Bissantz C.; Fasching B.; Gerebtzoff G.; Guba W.; Kansy M.; Migeon J.; Mohr S.; Peters J. U.; Tillier F.; Wyler R.; Lerner C.; Kramer C.; Richter H.; Roberts S. Safety Screening in Early Drug Discovery: An Optimized Assay Panel. J. Pharmacol Toxicol Methods 2019, 99, 106609 10.1016/j.vascn.2019.106609. 31284073

[ref43] Eurofins Discovery. https://www.eurofinsdiscovery.com/solution/safety-panels (accessed Sep 15, 2024).

[ref44] U.S. Food & Drug Administration. Guidance for Industry S7A Safety Pharmacology Studies for Human Pharmaceuticals. https://www.fda.gov/media/72033/download (accessed Jan 10, 2025).

[ref45] Klein M.; Busch M.; Friese-Hamim M.; Crosignani S.; Fuchss T.; Musil D.; Rohdich F.; Sanderson M. P.; Seenisamy J.; Walter-Bausch G.; Zanelli U.; Hewitt P.; Esdar C.; Schadt O. Structure-Based Optimization and Discovery of M3258, a Specific Inhibitor of the Immunoproteasome Subunit LMP7 (Β5i). J. Med. Chem. 2021, 64 (14), 10230–10245. 10.1021/acs.jmedchem.1c00604. 34228444

[ref46] Enomoto T.; Tatara A.; Goda M.; Nishizato Y.; Nishigori K.; Kitamura A.; Kamada M.; Taga S.; Hashimoto T.; Ikeda K.; Fujii Y. A Novel Phosphodiesterase 1 Inhibitor DSR-141562 Exhibits Efficacies in Animal Models for Positive, Negative, and Cognitive Symptoms Associated with Schizophrenia. J. Pharmacol Exp Ther 2019, 371 (3), 692–702. 10.1124/jpet.119.260869. 31578257

[ref47] Zhang H.; Cao X.; Tang M.; Zhong G.; Si Y.; Li H.; Zhu F.; Liao Q.; Li L.; Zhao J.; Feng J.; Li S.; Wang C.; Kaulich M.; Wang F.; Chen L.; Li L.; Xia Z.; Liang T.; Lu H.; Feng X. H.; Zhao B. A Subcellular Map of the Human Kinome. Elife 2021, 10. 10.7554/eLife.64943 PMC8175086 33988507

[ref48] Zhang M.; Liu Y.; Jang H.; Nussinov R. Strategy toward Kinase-Selective Drug Discovery. J. Chem. Theory Comput 2023, 19 (5), 1615–1628. 10.1021/acs.jctc.2c01171. 36815703 PMC10018734

[ref49] Palve V.; Liao Y.; Remsing Rix L. L.; Rix U. Turning Liabilities into Opportunities: Off-Target Based Drug Repurposing in Cancer. Semin Cancer Biol. 2021, 68, 209–229. 10.1016/j.semcancer.2020.02.003. 32044472 PMC7415607

[ref50] Cohen P.; Cross D.; Jänne P. A. Kinase Drug Discovery 20 Years after Imatinib: Progress and Future Directions. Nat. Rev. Drug Discov 2021, 20 (7), 551–569. 10.1038/s41573-021-00195-4. 34002056 PMC8127496

[ref51] Elkins J. M.; Fedele V.; Szklarz M.; Abdul Azeez K. R.; Salah E.; Mikolajczyk J.; Romanov S.; Sepetov N.; Huang X. P.; Roth B. L.; Al Haj Zen A.; Fourches D.; Muratov E.; Tropsha A.; Morris J.; Teicher B. A.; Kunkel M.; Polley E.; Lackey K. E.; Atkinson F. L.; Overington J. P.; Bamborough P.; Müller S.; Price D. J.; Willson T. M.; Drewry D. H.; Knapp S.; Zuercher W. J. Comprehensive Characterization of the Published Kinase Inhibitor Set. Nat. Biotechnol. 2016, 34 (1), 95–103. 10.1038/nbt.3374. 26501955

[ref52] Green J. R.; Mahalingaiah P. K. S.; Gopalakrishnan S. M.; Liguori M. J.; Mittelstadt S. W.; Blomme E. A. G.; Van Vleet T. R. Off-Target Pharmacological Activity at Various Kinases: Potential Functional and Pathological Side Effects. J. Pharmacol Toxicol Methods 2023, 123, 107468 10.1016/j.vascn.2023.107468. 37553032

[ref53] Reaction Biology Kinase Selectivity Profiling Panel. https://www.reactionbiology.com/services/kinase-discovery-services/ (accessed Sep 15, 2024).

[ref54] PanScreen. https://www.panscreen.ch (accessed Sep 15, 2024).

[ref55] Ietswaart R.; Arat S.; Chen A. X.; Farahmand S.; Kim B.; DuMouchel W.; Armstrong D.; Fekete A.; Sutherland J. J.; Urban L. Machine Learning Guided Association of Adverse Drug Reactions with in Vitro Target-Based Pharmacology. EBioMedicine 2020, 57, 102837 10.1016/j.ebiom.2020.102837. 32565027 PMC7379147

[ref56] Nussinov R.; Tsai C.-J. The Different Ways through Which Specificity Works in Orthosteric and Allosteric Drugs. Curr. Pharm. Des 2012, 18 (9), 1311 10.2174/138161212799436377. 22316155 PMC7458136

[ref57] The Toxicology in the 21st Century (Tox21) Program. https://tox21.gov (accessed Jan 10, 2025).

[ref58] Richard A. M.; Huang R.; Waidyanatha S.; Shinn P.; Collins B. J.; Thillainadarajah I.; Grulke C. M.; Williams A. J.; Lougee R. R.; Judson R. S.; Houck K. A.; Shobair M.; Yang C.; Rathman J. F.; Yasgar A.; Fitzpatrick S. C.; Simeonov A.; Thomas R. S.; Crofton K. M.; Paules R. S.; Bucher J. R.; Austin C. P.; Kavlock R. J.; Tice R. R. The Tox21 10K Compound Library: Collaborative Chemistry Advancing Toxicology. Chem. Res. Toxicol. 2021, 34 (2), 189–216. 10.1021/acs.chemrestox.0c00264. 33140634 PMC7887805

[ref59] Mayr A.; Klambauer G.; Unterthiner T.; Hochreiter S. DeepTox: Toxicity Prediction Using Deep Learning. Front Environ. Sci. 2016, 3, 167215 10.3389/fenvs.2015.00080.

[ref60] Huang R.; Xia M.; Nguyen D. T.; Zhao T.; Sakamuru S.; Zhao J.; Shahane S. A.; Rossoshek A.; Simeonov A. Tox21 Challenge to Build Predictive Models of Nuclear Receptor and Stress Response Pathways as Mediated by Exposure to Environmental Chemicals and Drugs. Front Environ. Sci. 2016, 3, 167167 10.3389/fenvs.2015.00085.

[ref61] Luo X.; Xu T.; Ngan D. K.; Xia M.; Zhao J.; Sakamuru S.; Simeonov A.; Huang R. Prediction of Chemical-Induced Acute Toxicity Using in Vitro Assay Data and Chemical Structure. Toxicol. Appl. Pharmacol. 2024, 492, 117098 10.1016/j.taap.2024.117098. 39251042 PMC11563913

[ref62] Kim D.; Jeong J.; Choi J. Identification of Optimal Machine Learning Algorithms and Molecular Fingerprints for Explainable Toxicity Prediction Models Using ToxCast/Tox21 Bioassay Data. ACS Omega 2024, 9 (36), 37934–37941. 10.1021/acsomega.4c04474. 39281924 PMC11391437

[ref63] Liu T.; Xu X.; Li J.; Bai M.; Zhu W.; Liu Y.; Liu S.; Zhao Z.; Li T.; Jiang N.; Bai Y.; Jin Q.; Zhang Y.; Zheng Y.; Zhou S.; Zhan S.; Sun Y.; Liang G.; Luo Y.; Chen X.; Guo H.; Yang R. ALOX5 Deficiency Contributes to Bladder Cancer Progression by Mediating Ferroptosis Escape. Cell Death Dis 2023, 14 ( (12), ). 10.1038/s41419-023-06333-7. PMC10703795 38062004

[ref64] Haeggström J. Z. Leukotriene Biosynthetic Enzymes as Therapeutic Targets. J. Clin Invest 2018, 128 (7), 2680–2690. 10.1172/JCI97945. 30108195 PMC6026001

[ref65] Bosley T. M.; Salih M. A.; Alorainy I. A.; Islam M. Z.; Oystreck D. T.; Suliman O. S. M.; Malki S. Al; Suhaibani A. H.; Khiari H.; Beckers S.; Van Wesenbeeck L.; Perdu B.; Aldrees A.; Elmalik S. A.; Van Hul W.; Abu-Amero K. K. The Neurology of Carbonic Anhydrase Type II Deficiency Syndrome. Brain 2011, 134 (12), 3502 10.1093/brain/awr302. 22120147

[ref66] Rosoff D. B.; Bell A. S.; Jung J.; Wagner J.; Mavromatis L. A.; Lohoff F. W. Mendelian Randomization Study of PCSK9 and HMG-CoA Reductase Inhibition and Cognitive Function. J. Am. Coll Cardiol 2022, 80 (7), 653–662. 10.1016/j.jacc.2022.05.041. 35953131

[ref67] Swerdlow D. I; Preiss D.; Kuchenbaecker K. B; Holmes M. V; Engmann J. E L; Shah T.; Sofat R.; Stender S.; Johnson P. C D; Scott R. A; Leusink M.; Verweij N.; Sharp S. J; Guo Y.; Giambartolomei C.; Chung C.; Peasey A.; Amuzu A.; Li K.; Palmen J.; Howard P.; Cooper J. A; Drenos F.; Li Y. R; Lowe G.; Gallacher J.; Stewart M. C W; Tzoulaki I.; Buxbaum S. G; van der A D. L; Forouhi N. G; Onland-Moret N C.; van der Schouw Y. T; Schnabel R. B; Hubacek J. A; Kubinova R.; Baceviciene M.; Tamosiunas A.; Pajak A.; Topor-Madry R.; Stepaniak U.; Malyutina S.; Baldassarre D.; Sennblad B.; Tremoli E.; de Faire U.; Veglia F.; Ford I.; Jukema J W.; Westendorp R. G J; de Borst G. J.; de Jong P. A; Algra A.; Spiering W.; der Zee A. H M.-v.; Klungel O. H; de Boer A.; Doevendans P. A; Eaton C. B; Robinson J. G; Duggan D.; Kjekshus J.; Downs J. R; Gotto A. M; Keech A. C; Marchioli R.; Tognoni G.; Sever P. S; Poulter N. R; Waters D. D; Pedersen T. R; Amarenco P.; Nakamura H.; McMurray J. J V; Lewsey J. D; Chasman D. I; Ridker P. M; Maggioni A. P; Tavazzi L.; Ray K. K; Seshasai S. R. K.; Manson J. E; Price J. F; Whincup P. H; Morris R. W; Lawlor D. A; Smith G. D.; Ben-Shlomo Y.; Schreiner P. J; Fornage M.; Siscovick D. S; Cushman M.; Kumari M.; Wareham N. J; Verschuren W M M.; Redline S.; Patel S. R; Whittaker J. C; Hamsten A.; Delaney J. A; Dale C.; Gaunt T. R; Wong A.; Kuh D.; Hardy R.; Kathiresan S.; Castillo B. A; van der Harst P.; Brunner E. J; Tybjaerg-Hansen A.; Marmot M. G; Krauss R. M; Tsai M.; Coresh J.; Hoogeveen R. C; Psaty B. M; Lange L. A; Hakonarson H.; Dudbridge F.; Humphries S. E; Talmud P. J; Kivimaki M.; Timpson N. J; Langenberg C.; Asselbergs F. W; Voevoda M.; Bobak M.; Pikhart H.; Wilson J. G; Reiner A. P; Keating B. J; Hingorani A. D; Sattar N. HMG-Coenzyme A Reductase Inhibition, Type 2 Diabetes, and Bodyweight: Evidence from Genetic Analysis and Randomised Trials. Lancet 2015, 385 (9965), 351–361. 10.1016/S0140-6736(14)61183-1. 25262344 PMC4322187

[ref68] Lagunas-Rangel F. A.; Liepinsh E.; Fredriksson R.; Alsehli A. M.; Williams M. J.; Dambrova M.; Jönsson J.; Schiöth H. B. Off-Target Effects of Statins: Molecular Mechanisms, Side Effects and the Emerging Role of Kinases. Br. J. Pharmacol. 2024, 181 (20), 3799 10.1111/bph.17309. 39180421

[ref69] Hidalgo M.; Eckhardt S. G. Development of Matrix Metalloproteinase Inhibitors in Cancer Therapy. J. Natl. Cancer Inst 2001, 93 (3), 178–193. 10.1093/jnci/93.3.178. 11158186

[ref70] Winer A.; Adams S.; Mignatti P. Matrix Metalloproteinase Inhibitors in Cancer Therapy: Turning Past Failures Into Future Successes. Mol. Cancer Ther 2018, 17 (6), 1147–1155. 10.1158/1535-7163.MCT-17-0646. 29735645 PMC5984693

[ref71] Soulaidopoulos S.; Terentes-Printzios D.; Ioakeimidis N.; Tsioufis K. P.; Vlachopoulos C. Long-Term Effects of Phosphodiesterase-5 Inhibitors on Cardiovascular Outcomes and Death: A Systematic Review and Meta-Analysis. Eur. Heart J. Cardiovasc Pharmacother 2024, 10 (5), 403–412. 10.1093/ehjcvp/pvae029. 38777751 PMC11323371

[ref72] Lui J. L.; Shaw N. M.; Abbasi B.; Hakam N.; Breyer B. N. Adverse Reactions of PDE5 Inhibitors: An Analysis of the World Health Organization Pharmacovigilance Database. Andrology 2023, 11 (7), 1408–1417. 10.1111/andr.13430. 36905319

[ref73] Tzoumas N.; Farrah T. E.; Dhaun N.; Webb D. J. Established and Emerging Therapeutic Uses of PDE Type 5 Inhibitors in Cardiovascular Disease. Br. J. Pharmacol. 2020, 177 (24), 5467–5488. 10.1111/bph.14920. 31721165 PMC7707100

[ref74] Tsai M.; Chrones L.; Xie J.; Gevorkyan H.; Macek T. A. A Phase 1 Study of the Safety, Tolerability, Pharmacokinetics, and Pharmacodynamics of TAK-063, a Selective PDE10A Inhibitor. Psychopharmacology (Berl) 2016, 233 (21–22), 3787–3795. 10.1007/s00213-016-4412-9. 27572830 PMC5063900

[ref75] Menniti F. S.; Chappie T. A.; Schmidt C. J. PDE10A Inhibitors-Clinical Failure or Window Into Antipsychotic Drug Action? Front Neurosci. 2021, 14. 10.3389/fnins.2020.600178 PMC7855852 33551724

[ref76] Geneviève D.; Proulle V.; Isidor B.; Bellais S.; Serre V.; Djouadi F.; Picard C.; Vignon-Savoye C.; Bader-Meunier B.; Blanche S.; De Vernejoul M. C.; Legeai-Mallet L.; Fischer A. M.; Le Merrer M.; Dreyfus M.; Gaussem P.; Munnich A.; Cormier-Daire V. Thromboxane Synthase Mutations in an Increased Bone Density Disorder (Ghosal Syndrome). Nat. Genet. 2008, 40 (3), 284–286. 10.1038/ng.2007.66. 18264100

[ref77] Vezza R.; Mezzasoma A. M.; Venditti G.; Gresele P. Prostaglandin Endoperoxides and Thromboxane A2 Activate the Same Receptor Isoforms in Human Platelets. Thromb Haemost 2002, 87 (1), 114–121. 10.1055/s-0037-1612953. 11848439

[ref78] Brown T. J.; Barrett N.; Meng H.; Ricciotti E.; McDonnell C.; Dancis A.; Qualtieri J.; FitzGerald G. A.; Cotter M.; Babushok D. V. Nonsteroidal Anti-Inflammatory Drugs as a Targeted Therapy for Bone Marrow Failure in Ghosal Hematodiaphyseal Dysplasia. Blood 2023, 141 (13), 1553–1559. 10.1182/blood.2022018667. 36574346 PMC10082374

