# Supplementary material for: The Missing Enzymes: A Call to Update Pharmacological Profiling Practices for Better Drug Safety Assessment
Source: J Med Chem. 2025 Apr 2;68(8):7854–65. doi: 10.1021/acs.jmedchem.4c02228 (PMC12035801; doi:10.1021/acs.jmedchem.4c02228)
Supplement: Supplementary file 1 — jm4c02228_si_001.pdf [file jm4c02228_si_001.pdf]

The Missing Enzymes: A Call to Update Pharmacological Profiling Practices for Better Drug Safety Assessment

Monika Maciag, Vardan T. Karamyan*

Department of Foundational Medical Sciences, William Beaumont School of Medicine,

Oakland University, Rochester, MI 48309, USA

Supporting Information

*** Correspondence should be addressed to**:

Vardan T. Karamyan, Pharm.D., Ph.D.

Oakland University William Beaumont School of Medicine

118 Library Drive, Rochester, MI 48309

United States of America

Phone: 248-370-4525

Email: vkaramyan@oakland.edu

Supporting Information S1: The list of shortlisted articles was compiled from two journals: the *Journal of Medicinal Chemistry* (JMC, with articles published between 2021 and 2023) and the *Journal of Pharmacology and Experimental Therapeutics* (JPET, with articles published between 2019 and 2023). These articles were used for pharmacological profiling data analysis.

(1) Baska, F.; Bozó, É.; Szeleczky, Z.; Szántó, G.; Vukics, K.; Szakács, Z.; Domány-Kovács, K.; Kurkó, D.; Vass, E.; Thán, M.; Vastag, M.; Temesvári, K.; Lévai, S.; Halász, A. S.; Szondiné Kordás, K.; Román, V.; Greiner, I.; Bata, I. Discovery and Characterization of RGH-122, a Potent, Selective, and Orally Bioavailable V1a Receptor Antagonist. *J. Med. Chem.* **2024**, *67* (1), 643–673.

(2) Thoma, G.; Markert, C.; Lueoend, R.; Miltz, W.; Spanka, C.; Bollbuck, B.; Wolf, R. M.; Srinivas, H.; Penno, C. A.; Kiffe, M.; Gajewska, M.; Bednarczyk, D.; Wieczorek, G.; Evans, A.; Beerli, C.; Röhn, T. A. Discovery of Amino Alcohols as Highly Potent, Selective, and Orally Efficacious Inhibitors of Leukotriene A4 Hydrolase. *J. Med. Chem.* **2023**, *66* (23), 16410–16425.

(3) Breinlinger, E.; Van Epps, S.; Friedman, M.; Argiriadi, M.; Chien, E.; Chhor, G.; Cowart, M.; Dunstan, T.; Graff, C.; Hardee, D.; Herold, J. M.; Little, A.; McCarthy, R.; Parmentier, J.; Perham, M.; Qiu, W.; Schrimpf, M.; Vargo, T.; Webster, M. P.; Wu, F.; Bennett, D.; Edmunds, J. Targeting the Tyrosine Kinase 2 (TYK2) Pseudokinase Domain: Discovery of the Selective TYK2 Inhibitor ABBV-712. *J. Med. Chem.* **2023**, *66* (20), 14335–14356.

(4) Zhu, G.; Li, J.; Lin, X.; Zhang, Z.; Hu, T.; Huo, S.; Li, Y. Discovery of a Novel Ketohexokinase Inhibitor with Improved Drug Distribution in Target Tissue for the Treatment of Fructose Metabolic Disease. *J. Med. Chem.* **2023**, *66* (19), 13501–13515.

(5) Zheng, Y.; van den Kerkhof, M.; van der Meer, T.; Gul, S.; Kuzikov, M.; Ellinger, B.; de Esch, I. J. P.; Siderius, M.; Matheeussen, A.; Maes, L.; Sterk, G. J.; Caljon, G.; Leurs, R. Discovery of 5-Phenylpyrazolopyrimidinone Analogs as Potent Antitrypanosomal Agents with In Vivo Efficacy. *J. Med. Chem.* **2023**, *66* (15), 10252–10264.

(6) Shukla, M. R.; Sadasivam, G.; Sarde, A.; Sayyed, M.; Pachpute, V.; Phadtare, R.; Walke, N.; Chaudhari, V. D.; Loriya, R.; Khan, T.; Gote, G.; Pawar, C.; Tryambake, M.; Mahajan, N.; Gandhe, A.; Sabde, S.; Pawar, S.; Patil, V.; Modi, D.; Mehta, M.; Nigade, P.; Modak, V.; Ghodke, R.; Narasimham, L.; Bhonde, M.; Gundu, J.; Goel, R.; Shah, C.; Kulkarni, S.; Sharma, S.; Bakhle, D.; Kamboj, R. K.; Palle, V. P. Discovery of LNP1892: A Precision Calcimimetic for the Treatment of Secondary Hyperparathyroidism. *J. Med. Chem.* **2023**, *66* (14), 9418–9444.

(7) Le, K.; Soth, M. J.; Cross, J. B.; Liu, G.; Ray, W. J.; Ma, J.; Goodwani, S. G.; Acton, P. J.; Buggia-Prevot, V.; Akkermans, O.; Barker, J.; Conner, M. L.; Jiang, Y.; Liu, Z.; McEwan, P.; Warner-

Schmidt, J.; Xu, A.; Zebisch, M.; Heijnen, C. J.; Abrahams, B.; Jones, P. Discovery of IACS-52825, a Potent and Selective DLK Inhibitor for Treatment of Chemotherapy-Induced Peripheral Neuropathy. *J. Med. Chem.* **2023**, *66* (14), 9954–9971.

(8) Pasqua, A. E.; Sharp, S. Y.; Chessum, N. E. A.; Hayes, A.; Pellegrino, L.; Tucker, M. J.; Miah, A.; Wilding, B.; Evans, L. E.; Rye, C. S.; Mok, N. Y.; Liu, M.; Henley, A. T.; Gowan, S.; De Billy, E.; te Poele, R.; Powers, M.; Eccles, S. A.; Clarke, P. A.; Raynaud, F. I.; Workman, P.; Jones, K.; Cheeseman, M. D. HSF1 Pathway Inhibitor Clinical Candidate (CCT361814/NXP800) Developed from a Phenotypic Screen as a Potential Treatment for Refractory Ovarian Cancer and Other Malignancies. *J. Med. Chem.* **2023**, *66* (8), 5907–5936.

(9) Ishikawa, T.; Hara, H.; Kawano, A.; Tohyama, K.; Kajita, Y.; Miyanohana, Y.; Koike, T.; Kimura, H. TAK-994, a Novel Orally Available Brain-Penetrant Orexin 2 Receptor-Selective Agonist, Suppresses Fragmentation of Wakefulness and Cataplexy-Like Episodes in Mouse Models of Narcolepsy. *J. Pharmacol. Exp. Ther.* **2023**, *385* (3), 193–204.

(10) Meibom, D.; Meyer, J.; von Buehler, C. J.; Collins, K. D.; Maassen, S.; Gericke, K. M.; Hüser, J.; Mittendorf, J.; Ortega Hernandez, N.; Schamberger, J.; Stampfuss, J.; Straub, A.; Torge, A.; Witowski, N.; Wunder, F. BAY-6096: A Potent, Selective, and Highly Water-Soluble Adrenergic A2B Antagonist. *J. Med. Chem.* **2023**, *66* (7), 4659–4670.

(11) Göricke, F.; Vu, V.; Smith, L.; Scheib, U.; Böhm, R.; Akkilic, N.; Wohlfahrt, G.; Weiske, J.; Bömer, U.; Brzezinka, K.; Lindner, N.; Lienau, P.; Gradl, S.; Beck, H.; Brown, P. J.; Santhakumar, V.; Vedadi, M.; Barsyte-Lovejoy, D.; Arrowsmith, C. H.; Schmees, N.; Petersen, K. Discovery and Characterization of BAY-805, a Potent and Selective Inhibitor of Ubiquitin-Specific Protease USP21. *J. Med. Chem.* **2023**, *66* (5), 3431–3447.

(12) Garnsey, M. R.; Smith, A. C.; Polivkova, J.; Arons, A. L.; Bai, G.; Blakemore, C.; Boehm, M.; Buzon, L. M.; Campion, S. N.; Cerny, M.; Chang, S. C.; Coffman, K.; Farley, K. A.; Fonseca, K. R.; Ford, K. K.; Garren, J.; Kong, J. X.; Koos, M. R. M.; Kung, D. W.; Lian, Y.; Li, M. M.; Li, Q.; Martinez-Alsina, L. A.; O'Connor, R.; Ogilvie, K.; Omoto, K.; Raymer, B.; Reese, M. R.; Ryder, T.; Samp, L.; Stevens, K. A.; Widlicka, D. W.; Yang, Q.; Zhu, K.; Fortin, J. P.; Sammons, M. F. Discovery of the Potent and Selective MC4R Antagonist PF-07258669 for the Potential Treatment of Appetite Loss. *J. Med. Chem.* **2023**, *66* (5), 3195–3211.

(13) Thamm, S.; Willwacher, M. K.; Aspnes, G. E.; Bretschneider, T.; Brown, N. F.; Buschbom-Helmke, S.; Fox, T.; Gargano, E. M.; Grabowski, D.; Hoenke, C.; Matera, D.; Mueck, K.; Peters, S.; Reindl, S.; Riether, D.; Schmid, M.; Tautermann, C. S.; Teitelbaum, A. M.; Trünkle, C.; Veser, T.; Winter,

M.; Wortmann, L. Discovery of a Novel Potent and Selective HSD17B13 Inhibitor, BI-3231, a Well-Characterized Chemical Probe Available for Open Science. *J. Med. Chem.* **2023**, *66* (4), 2832–2850.

(14) Deschaine, S. L.; Hedegaard, M. A.; Pince, C. L.; Farokhnia, M.; Moose, J. E.; Stock, I. A.; Adusumalli, S.; Akhlaghi, F.; Hougland, J. L.; Sulima, A.; Rice, K. C.; Koob, G. F.; Vendruscolo, L. F.; Holst, B.; Leggio, L. Initial Pharmacological Characterization of a Major Hydroxy Metabolite of PF-5190457: Inverse Agonist Activity of PF-6870961 at the Ghrelin Receptor. *J. Pharmacol. Exp. Ther.* **2023**, *386* (2), 117–128.

(15) Mesch, S.; Walter, D.; Laux-Biehlmann, A.; Basting, D.; Flanagan, S.; Miyatake Ondozabal, H.; Bäurle, S.; Pearson, C.; Jenkins, J.; Elves, P.; Hess, S.; Coelho, A. M.; Rotgeri, A.; Bothe, U.; Nawaz, S.; Zollner, T. M.; Steinmeyer, A. Discovery of BAY-390, a Selective CNS Penetrant Chemical Probe as Transient Receptor Potential Ankyrin 1 (TRPA1) Antagonist. *J. Med. Chem.* **2023**, *66* (2), 1583–1600.

(16) Burnett, G. L.; Yang, Y. C.; Aggen, J. B.; Pitzen, J.; Gliedt, M. K.; Semko, C. M.; Marquez, A.; Evans, J. W.; Wang, G.; Won, W. S.; Tomlinson, A. C. A.; Kiss, G.; Tzitzilonis, C.; Thottumkara, A. P.; Cregg, J.; Mellem, K. T.; Choi, J. S.; Lee, J. C.; Zhao, Y.; Lee, B. J.; Meyerowitz, J. G.; Knox, J. E.; Jiang, J.; Wang, Z.; Wildes, D.; Wang, Z.; Singh, M.; Smith, J. A. M.; Gill, A. L. Discovery of RMC-5552, a Selective Bi-Steric Inhibitor of MTORC1, for the Treatment of MTORC1-Activated Tumors. *J. Med. Chem.* **2023**, *66* (1), 149–169.

(17) Meibom, D.; Micus, S.; Andreevski, A. L.; Anlauf, S.; Bogner, P.; Von Buehler, C. J.; Dieskau, A. P.; Dreher, J.; Eitner, F.; Fliegner, D.; Follmann, M.; Gericke, K. M.; Maassen, S.; Meyer, J.; Schlemmer, K. H.; Steuber, H.; Tersteegen, A.; Wunder, F. BAY-7081: A Potent, Selective, and Orally Bioavailable Cyanopyridone-Based PDE9A Inhibitor. *J. Med. Chem.* **2022**, *65* (24), 16420–16431.

(18) Hanan, E. J.; Braun, M. G.; Heald, R. A.; Macleod, C.; Chan, C.; Clausen, S.; Edgar, K. A.; Eigenbrot, C.; Elliott, R.; Endres, N.; Friedman, L. S.; Gogol, E.; Gu, X. H.; Thibodeau, R. H.; Jackson, P. S.; Kiefer, J. R.; Knight, J. D.; Nannini, M.; Narukulla, R.; Pace, A.; Pang, J.; Purkey, H. E.; Salphati, L.; Sampath, D.; Schmidt, S.; Sideris, S.; Song, K.; Sujatha-Bhaskar, S.; Ultsch, M.; Wallweber, H.; Xin, J.; Yeap, S.; Young, A.; Zhong, Y.; Staben, S. T. Discovery of GDC-0077 (Inavolisib), a Highly Selective Inhibitor and Degrader of Mutant PI3Kα. *J. Med. Chem.* **2022**, *65* (24), 16589–16621.

(19) Miller, M.; Rossetti, T.; Ferreira, J.; Ghanem, L.; Balbach, M.; Kaur, N.; Levin, L. R.; Buck, J.; Kehr, M.; Coquille, S.; Van Den Heuvel, J.; Steegborn, C.; Fushimi, M.; Finkin-Groner, E.; Myers, R. W.; Kargman, S.; Liverton, N. J.; Huggins, D. J.; Meinke, P. T. Design, Synthesis, and Pharmacological Evaluation of Second-Generation Soluble Adenylyl Cyclase (SAC, ADCY10) Inhibitors with Slow Dissociation Rates. *J. Med. Chem.* **2022**, *65* (22), 15208–15226.

(20) Wang, F. C.; Peng, B.; Ren, T. T.; Liu, S. P.; Du, J. R.; Chen, Z. H.; Zhang, T. T.; Gu, X.; Li, M.; Cao, S. L.; Xu, X. A 1,2,3-Triazole Derivative of Quinazoline Exhibits Antitumor Activity by Tethering RNF168 to SQSTM1/P62. *J. Med. Chem.* **2022**, *65* (22), 15028–15047.

(21) Futatsugi, K.; Cabral, S.; Kung, D. W.; Huard, K.; Lee, E.; Boehm, M.; Bauman, J.; Clark, R. W.; Coffey, S. B.; Crowley, C.; Dechert-Schmitt, A. M.; Dowling, M. S.; Dullea, R.; Gosset, J. R.; Kalgutkar, A. S.; Kou, K.; Li, Q.; Lian, Y.; Loria, P. M.; Londregan, A. T.; Niosi, M.; Orozco, C.; Pettersen, J. C.; Pfefferkorn, J. A.; Polivkova, J.; Ross, T. T.; Sharma, R.; Stock, I. A.; Tesz, G.; Wisniewska, H.; Goodwin, B.; Price, D. A. Discovery of Ervogastat (PF-06865571): A Potent and Selective Inhibitor of Diacylglycerol Acyltransferase 2 for the Treatment of Non-Alcoholic Steatohepatitis. *J. Med. Chem.* **2022**, *65* (22), 15000–15013.

(22) Barrows, R. D.; Jeffries, D. E.; Vishe, M.; Tukachinsky, H.; Zheng, S. L.; Li, F.; Ma, Z.; Li, X.; Jin, S.; Song, H.; Zhang, R.; Zhang, S.; Ni, J.; Luan, H.; Wen, L.; Rongshan, Y.; Ying, C.; Shair, M. D. Potent Uncompetitive Inhibitors of Nicotinamide N-Methyltransferase (NNMT) as In Vivo Chemical Probes. *J. Med. Chem.* **2022**, *65* (21), 14642–14654.

(23) Günther, J.; Hillig, R. C.; Zimmermann, K.; Kaulfuss, S.; Lemos, C.; Nguyen, D.; Rehwinkel, H.; Habgood, M.; Lechner, C.; Neuhaus, R.; Ganzer, U.; Drewes, M.; Chai, J.; Bouché, L. BAY-069, a Novel (Trifluoromethyl)Pyrimidinedione-Based BCAT1/2 Inhibitor and Chemical Probe. *J. Med. Chem.* **2022**, *65* (21), 14366–14390.

(24) Lowe, M. A.; Cardenas, A.; Valentin, J. P.; Zhu, Z.; Abendroth, J.; Castro, J. L.; Class, R.; Delaunois, A.; Fleurance, R.; Gerets, H.; Gryshkova, V.; King, L.; Lorimer, D. D.; Maccoss, M.; Rowley, J. H.; Rosseels, M. L.; Royer, L.; Taylor, R. D.; Wong, M.; Zaccheo, O.; Chavan, V. P.; Ghule, G. A.; Tapkir, B. K.; Burrows, J. N.; Duffey, M.; Rottmann, M.; Wittlin, S.; Angulo-Barturen, I.; Jiménez-Díaz, M. B.; Striepen, J.; Fairhurst, K. J.; Yeo, T.; Fidock, D. A.; Cowman, A. F.; Favuzza, P.; Crespo-Fernandez, B.; Gamo, F. J.; Goldberg, D. E.; Soldati-Favre, D.; Laleu, B.; De Haro, T. Discovery and Characterization of Potent, Efficacious, Orally Available Antimalarial Plasmepsin X Inhibitors and Preclinical Safety Assessment of UCB7362. *J. Med. Chem.* **2022**, *65* (20), 14121–14143.

(25) Obst-Sander, U.; Ricci, A.; Kuhn, B.; Friess, T.; Koldewey, P.; Kuglstatter, A.; Hewings, D.; Goergler, A.; Steiner, S.; Rueher, D.; Imhoff, M. P.; Raschetti, N.; Marty, H. P.; Dietzig, A.; Rynn, C.; Ehler, A.; Burger, D.; Kornacker, M.; Schaffland, J. P.; Herting, F.; Pao, W.; Bischoff, J. R.; Martoglio, B.; Alice Nagel, Y.; Jaeschke, G. Discovery of Novel Allosteric EGFR L858R Inhibitors for the Treatment of Non-Small-Cell Lung Cancer as a Single Agent or in Combination with Osimertinib. *J. Med. Chem.* **2022**, *65* (19), 13052–13073.

(26) Arici, M.; Ferrandi, M.; Barassi, P.; Hsu, S. C.; Torre, E.; Luraghi, A.; Ronchi, C.; Chang, G. J.; Peri, F.; Ferrari, P.; Bianchi, G.; Rocchetti, M.; Zaza, A. Istaroxime Metabolite PST3093 Selectively Stimulates SERCA2a and Reverses Disease-Induced Changes in Cardiac Function. *J. Pharmacol. Exp. Ther.* **2023**, *384* (1), 231–244.

(27) Zetterberg, F. R.; Mackinnon, A.; Brimert, T.; Gravelle, L.; Johnsson, R. E.; Kahl-Knutson, B.; Leffler, H.; Nilsson, U. J.; Pedersen, A.; Peterson, K.; Roper, J. A.; Schambye, H.; Slack, R. J.; Tantawi, S. Discovery and Optimization of the First Highly Effective and Orally Available Galectin-3 Inhibitors for Treatment of Fibrotic Disease. *J. Med. Chem.* **2022**, *65* (19), 12626–12638.

(28) Ketcham, J. M.; Haling, J.; Khare, S.; Bowcut, V.; Briere, D. M.; Burns, A. C.; Gunn, R. J.; Ivetac, A.; Kuehler, J.; Kulyk, S.; Laguer, J.; Lawson, J. D.; Moya, K.; Nguyen, N.; Rahbaek, L.; Saechao, B.; Smith, C. R.; Sudhakar, N.; Thomas, N. C.; Vegar, L.; Vanderpool, D.; Wang, X.; Yan, L.; Olson, P.; Christensen, J. G.; Marx, M. A. Design and Discovery of MRTX0902, a Potent, Selective, Brain-Penetrant, and Orally Bioavailable Inhibitor of the SOS1:KRAS Protein-Protein Interaction. *J. Med. Chem.* **2022**, *65* (14), 9678–9690.

(29) Hill, M. D.; Blanco, M. J.; Salituro, F. G.; Bai, Z.; Beckley, J. T.; Ackley, M. A.; Dai, J.; Doherty, J. J.; Harrison, B. L.; Hoffmann, E. C.; Kazdoba, T. M.; Lanzetta, D.; Lewis, M.; Quirk, M. C.; Robichaud, A. J. SAGE-718: A First-in-Class N-Methyl-d-Aspartate Receptor Positive Allosteric Modulator for the Potential Treatment of Cognitive Impairment. *J. Med. Chem.* **2022**, *65* (13), 9063–9075.

(30) Takeuchi, T.; Hayashi, M.; Tamita, T.; Nomura, Y.; Kojima, N.; Mitani, A.; Takeda, T.; Hitaka, K.; Kato, Y.; Kamitani, M.; Mima, M.; Toki, H.; Ohkubo, M.; Nozoe, A.; Kakinuma, H. Discovery of Aryloxyphenyl-Heptapeptide Hybrids as Potent and Selective Matrix Metalloproteinase-2 Inhibitors for the Treatment of Idiopathic Pulmonary Fibrosis. *J. Med. Chem.* **2022**, *65* (12), 8493–8510.

(31) Willis, N. J.; Mahy, W.; Sipthorp, J.; Zhao, Y.; Woodward, H. L.; Atkinson, B. N.; Bayle, E. D.; Svensson, F.; Frew, S.; Jeganathan, F.; Monaghan, A.; Benvegnù, S.; Jolly, S.; Vecchia, L.; Ruza, R. R.; Kjær, S.; Howell, S.; Snijders, A. P.; Bictash, M.; Salinas, P. C.; Vincent, J. P.; Jones, E. Y.;

Whiting, P.; Fish, P. V. Design of a Potent, Selective, and Brain-Penetrant Inhibitor of Wnt-Deactivating Enzyme Notum by Optimization of a Crystallographic Fragment Hit. *J. Med. Chem.* **2022**, *65* (10), 7212–7230.

(32) Decara, J. M.; Vázquez-Villa, H.; Brea, J.; Alonso, M.; Srivastava, R. K.; Orio, L.; Alén, F.; Suárez, J.; Baixeras, E.; García-Cárceles, J.; Escobar-Peña, A.; Lutz, B.; Rodríguez, R.; Codesido, E.; Garcia-Ladona, F. J.; Bennett, T. A.; Ballesteros, J. A.; Cruces, J.; Loza, M. I.; Benhamú, B.; Rodríguez De Fonseca, F.; López-Rodríguez, M. L. Discovery of V-0219: A Small-Molecule Positive Allosteric Modulator of the Glucagon-Like Peptide-1 Receptor toward Oral Treatment for “Diabesity.” *J. Med. Chem.* **2022**, *65* (7), 5449–5461.

(33) Scott, J. A.; Soto-Velasquez, M.; Hayes, M. P.; Lavigne, J. E.; Miller, H. R.; Kaur, J.; Ejendal, K. F. K.; Watts, V. J.; Flaherty, D. P. Optimization of a Pyrimidinone Series for Selective Inhibition of Ca2+/Calmodulin-Stimulated Adenylyl Cyclase 1 Activity for the Treatment of Chronic Pain. *J. Med. Chem.* **2022**, *65* (6), 4667–4686.

(34) Wen, Z.; Salmaso, V.; Jung, Y. H.; Phung, N. B.; Gopinatth, V.; Shah, Q.; Patterson, A. T.; Randle, J. C. R.; Chen, Z.; Salvemini, D.; Lieberman, D. I.; Whitehead, G. S.; Karcz, T. P.; Cook, D. N.; Jacobson, K. A. Bridged Piperidine Analogues of a High Affinity Naphthalene-Based P2Y14R Antagonist. *J. Med. Chem.* **2022**, *65* (4), 3434–3459.

(35) Begnini, F.; Geschwindner, S.; Johansson, P.; Wissler, L.; Lewis, R. J.; Danelius, E.; Luttens, A.; Matricon, P.; Carlsson, J.; Lenders, S.; König, B.; Friedel, A.; Sjö, P.; Schiesser, S.; Kihlberg, J. Importance of Binding Site Hydration and Flexibility Revealed When Optimizing a Macrocyclic Inhibitor of the Keap1-Nrf2 Protein-Protein Interaction. *J. Med. Chem.* **2022**, *65* (4), 3473–3517.

(36) Humphreys, P. G.; Atkinson, S. J.; Bamborough, P.; Bit, R. A.; Chung, C. W.; Craggs, P. D.; Cutler, L.; Davis, R.; Ferrie, A.; Gong, G.; Gordon, L. J.; Gray, M.; Harrison, L. A.; Hayhow, T. G.; Haynes, A.; Henley, N.; Hirst, D. J.; Holyer, I. D.; Lindon, M. J.; Lovatt, C.; Lugo, D.; McCleary, S.; Molnar, J.; Osmani, Q.; Patten, C.; Preston, A.; Rioja, I.; Seal, J. T.; Smithers, N.; Sun, F.; Tang, D.; Taylor, S.; Theodoulou, N. H.; Thomas, C.; Watson, R. J.; Wellaway, C. R.; Zhu, L.; Tomkinson, N. C. O.; Prinjha, R. K. Design, Synthesis, and Characterization of I-BET567, a Pan-Bromodomain and Extra Terminal (BET) Bromodomain Oral Candidate. *J. Med. Chem.* **2022**, *65* (3), 2262–2287.

(37) Liu, Z.; Li, Y.; Chen, H.; Lai, H. T.; Wang, P.; Wu, S. Y.; Wold, E. A.; Leonard, P. G.; Joseph, S.; Hu, H.; Chiang, C. M.; Brasier, A. R.; Tian, B.; Zhou, J. Discovery, X-Ray Crystallography, and Anti-Inflammatory Activity of Bromodomain-Containing Protein 4 (BRD4) BD1 Inhibitors Targeting a Distinct New Binding Site. *J. Med. Chem.* **2022**, *65* (3), 2388–2408.

(38) Lovelock, D. F.; Nguyen, T.; Van Voorhies, K.; Zhang, Y.; Besheer, J. RTICBM-74 Is a Brain-Penetrant Cannabinoid Receptor Subtype 1 Allosteric Modulator That Reduces Alcohol Intake in Rats. *J. Pharmacol. Exp. Ther.* 2022, *380* (3), 153–161. https://doi.org/10.1124/JPET.121.000919.

(39) Tahtouh, T.; Durieu, E.; Villiers, B.; Bruyère, C.; Nguyen, T. L.; Fant, X.; Ahn, K. H.; Khurana, L.; Deau, E.; Lindberg, M. F.; Sévère, E.; Miege, F.; Roche, D.; Limanton, E.; L'Helgoual'ch, J. M.; Burgy, G.; Guiheneuf, S.; Herault, Y.; Kendall, D. A.; Carreaux, F.; Bazureau, J. P.; Meijer, L. Structure-Activity Relationship in the Leucettine Family of Kinase Inhibitors. *J. Med. Chem.* **2022**, *65* (2), 1396–1417.

(40) Boyd, M. J.; Collier, P. N.; Clark, M. P.; Deng, H.; Kesavan, S.; Ronkin, S. M.; Waal, N.; Wang, J.; Cao, J.; Li, P.; Come, J.; Davies, I.; Duffy, J. P.; Cochran, J. E.; Court, J. J.; Chandupatla, K.; Jackson, K. L.; Maltais, F.; O'Dowd, H.; Boucher, C.; Considine, T.; Taylor, W. P.; Gao, H.; Chakilam, A.; Engtrakul, J.; Crawford, D.; Doyle, E.; Phillips, J.; Kemper, R.; Swett, R.; Empfield, J.; Bunnage, M. E.; Charifson, P. S.; Magavi, S. S. Discovery of Novel, Orally Bioavailable Pyrimidine Ether-Based Inhibitors of ELOVL1. *J. Med. Chem.* **2021**, *64* (24), 17777–17794.

(41) Come, J. H.; Senter, T. J.; Clark, M. P.; Court, J. J.; Gale-Day, Z.; Gu, W.; Krueger, E.; Liang, J.; Morris, M.; Nanthakumar, S.; O'Dowd, H.; Maltais, F.; Iyer, G.; Andreassi, J.; Boucher, C.; Considine, T.; Moody, C. S.; Taylor, W.; Mohanty, A. K.; Huang, Y.; Zuccola, H.; Coll, J.; Bonanno, K. C.; Gagnon, K. J.; Gan, L.; Lu, F.; Gao, H.; Chakilam, A.; Engtrakul, J.; Song, B.; Crawford, D.; Doyle, E.; Kramer, T.; Vought, B.; Phillips, J.; Kemper, R.; Sanders, M.; Swett, R.; Furey, B.; Winquist, R.; Bunnage, M. E.; Jackson, K. L.; Charifson, P. S.; Magavi, S. S. Discovery and Optimization of Pyrazole Amides as Inhibitors of ELOVL1. *J. Med. Chem.* **2021**, *64* (24).

(42) Hopkins, B. T.; Bame, E.; Bajrami, B.; Black, C.; Bohnert, T.; Boiselle, C.; Burdette, D.; Burns, J. C.; Delva, L.; Donaldson, D.; Grater, R.; Gu, C.; Hoemberger, M.; Johnson, J.; Kapadnis, S.; King, K.; Lulla, M.; Ma, B.; Marx, I.; Magee, T.; Meissner, R.; Metrick, C. M.; Mingueneau, M.; Murugan, P.; Otipoby, K. L.; Polack, E.; Poreci, U.; Prince, R.; Roach, A. M.; Rowbottom, C.; Santoro, J. C.; Schroeder, P.; Tang, H.; Tien, E.; Zhang, F.; Lyssikatos, J. Discovery and Preclinical Characterization of BIIB091, a Reversible, Selective BTK Inhibitor for the Treatment of Multiple Sclerosis. *J. Med. Chem.* **2022**, *65* (2), 1206–1224.

(43) Mowbray, C. E.; Braillard, S.; Glossop, P. A.; Whitlock, G. A.; Jacobs, R. T.; Speake, J.; Pandi, B.; Nare, B.; Maes, L.; Yardley, V.; Freund, Y.; Wall, R. J.; Carvalho, S.; Bello, D.; Van Den Kerkhof, M.; Caljon, G.; Gilbert, I. H.; Corpas-Lopez, V.; Lukac, I.; Patterson, S.; Zuccotto, F.; Wyllie, S. DNDI-6148: A Novel Benzoxaborole Preclinical Candidate for the Treatment of Visceral Leishmaniasis. *J. Med. Chem.* **2021**, *64* (21), 16159–16176.

(44) Collibee, S. E.; Bergnes, G.; Chuang, C.; Ashcraft, L.; Gardina, J.; Garard, M.; Jamison, C. R.; Lu, K.; Lu, P. P.; Muci, A.; Romero, A.; Valkevich, E.; Wang, W.; Warrington, J.; Yao, B.; Durham, N.; Hartman, J.; Marquez, A.; Hinken, A.; Schaletzky, J.; Xu, D.; Hwee, D. T.; Morgans, D.; Malik, F. I.; Morgan, B. P. Discovery of Reldesemtiv, a Fast Skeletal Muscle Troponin Activator for the Treatment of Impaired Muscle Function. *J. Med. Chem.* **2021**, *64* (20), 14930–14941.

(45) Lu, Y.; Vibhute, S.; Li, L.; Okumu, A.; Ratigan, S. C.; Nolan, S.; Papa, J. L.; Mann, C. A.; English, A.; Chen, A.; Seffernick, J. T.; Koci, B.; Duncan, L. R.; Roth, B.; Cummings, J. E.; Slayden, R. A.; Lindert, S.; McElroy, C. A.; Wozniak, D. J.; Yalowich, J.; Mitton-Fry, M. J. Optimization of TopoIV Potency, ADMET Properties, and HERG Inhibition of 5-Amino-1,3-Dioxane-Linked Novel Bacterial Topoisomerase Inhibitors: Identification of a Lead with In Vivo Efficacy against MRSA. *J. Med. Chem.* **2021**, *64* (20), 15214–15249.

(46) Rombouts, F. J. R.; Kusakabe, K. I.; Alexander, R.; Austin, N.; Borghys, H.; De Cleyn, M.; Dhuyvetter, D.; Gijsen, H. J. M.; Hrupka, B.; Jacobs, T.; Jerhaoui, S.; Lammens, L.; Leclercq, L.; Tsubone, K.; Ueno, T.; Morimoto, K.; Einaru, S.; Sumiyoshi, H.; Van Den Bergh, A.; Vos, A.; Surkyn, M.; Teisman, A.; Moechars, D. JNJ-67569762, A 2-Aminotetrahydropyridine-Based Selective BACE1 Inhibitor Targeting the S3 Pocket: From Discovery to Clinical Candidate. *J. Med. Chem.* **2021**, *64* (19), 14175–14191.

(47) Matsuda, S.; Hattori, Y.; Matsumiya, K.; McQuade, P.; Yamashita, T.; Aida, J.; Sandiego, C. M.; Gouasmat, A.; Carroll, V. M.; Barret, O.; Tamagnan, G.; Koike, T.; Kimura, H. Design, Synthesis, and Evaluation of [18F]T-914 as a Novel Positron-Emission Tomography Tracer for Lysine-Specific Demethylase 1. *J. Med. Chem.* **2021**, *64* (17), 12680–12690.

(48) Zajdel, P.; Grychowska, K.; Mogilski, S.; Kurczab, R.; Satała, G.; Bugno, R.; Kos, T.; Gołębiowska, J.; Malikowska-Racia, N.; Nikiforuk, A.; Chaumont-Dubel, S.; Bantreil, X.; Pawłowski, M.; Martinez, J.; Subra, G.; Lamaty, F.; Marin, P.; Bojarski, A. J.; Popik, P. Structure-Based Design and Optimization of FPPQ, a Dual-Acting 5-HT3 and 5-HT6 Receptor Antagonist with Antipsychotic and Procognitive Properties. *J. Med. Chem.* **2021**, *64* (18), 13279–13298.

(49) Bellenie, B. R.; Hall, E.; Bruce, I.; Spendiff, M.; Culshaw, A.; McDonald, S.; Ambarkhane, A.; Chinn, C.; Thomas, M.; Rosner, E.; Bracher, M.; Nicklin, P.; Marshall, S.; Coote, J.; Cullen, E.; Tessier, C.; Wuersch, K.; Lal, A.; Wallis, G.; Hollingworth, G. J.; Neef, J. Discovery and Toxicological Profiling of Aminopyridines as Orally Bioavailable Selective Inhibitors of PI3-Kinase γ. *J. Med. Chem.* **2021**, *64* (16), 12304–12321.

(50) Liu, L.; Johnson, P. D.; Prime, M. E.; Khetarpal, V.; Lee, M. R.; Brown, C. J.; Chen, X.; Clark-Frew, D.; Coe, S.; Conlon, M.; Davis, R.; Ensor, S.; Esposito, S.; Moren, A. F.; Gai, X.; Green, S.; Greenaway, C.; Haber, J.; Halldin, C.; Hayes, S.; Herbst, T.; Herrmann, F.; Heßmann, M.; Hsai, M. M.; Kotey, A.; Mangette, J. E.; Mills, M. R.; Monteagudo, E.; Nag, S.; Nibbio, M.; Orsatti, L.; Schaertl, S.; Scheich, C.; Sproston, J.; Stepanov, V.; Varnäs, K.; Varrone, A.; Wityak, J.; Mrzljak, L.; Munoz-Sanjuan, I.; Bard, J. A.; Dominguez, C. [11C]CHDI-626, a PET Tracer Candidate for Imaging Mutant Huntingtin Aggregates with Reduced Binding to AD Pathological Proteins. *J. Med. Chem.* **2021**, *64* (16), 12003–12021.

(51) Oboh, E.; Schubert, T. J.; Teixeira, J. E.; Stebbins, E. E.; Miller, P.; Philo, E.; Thakellapalli, H.; Campbell, S. D.; Griggs, D. W.; Huston, C. D.; Meyers, M. J. Optimization of the Urea Linker of Triazolopyridazine MMV665917 Results in a New Anticryptosporidial Lead with Improved Potency and Predicted HERG Safety Margin. *J. Med. Chem.* **2021**, *64* (15), 11729–11745.

(52) Wu, L.; Zhang, C.; He, C.; Qian, D.; Lu, L.; Sun, Y.; Xu, M.; Zhuo, J.; Liu, P. C. C.; Klabe, R.; Wynn, R.; Covington, M.; Gallagher, K.; Leffet, L.; Bowman, K.; Diamond, S.; Koblish, H.; Zhang, Y.; Soloviev, M.; Hollis, G.; Burn, T. C.; Scherle, P.; Yeleswaram, S.; Huber, R.; Yao, W. Discovery of Pemigatinib: A Potent and Selective Fibroblast Growth Factor Receptor (FGFR) Inhibitor. *J. Med. Chem.* **2021**, *64* (15), 10666–10679.

(53) Reichard, H. A.; Schiffer, H. H.; Monenschein, H.; Atienza, J. M.; Corbett, G.; Skaggs, A. W.; Collia, D. R.; Ray, W. J.; Serrats, J.; Bliesath, J.; Kaushal, N.; Lam, B. P.; Amador-Arjona, A.; Rahbaek, L.; McConn, D. J.; Mulligan, V. J.; Brice, N.; Gaskin, P. L. R.; Cilia, J.; Hitchcock, S. Discovery of TAK-041: A Potent and Selective GPR139 Agonist Explored for the Treatment of Negative Symptoms Associated with Schizophrenia. *J. Med. Chem.* **2021**, *64* (15), 11527–11542.

(54) Seal, J. T.; Atkinson, S. J.; Bamborough, P.; Bassil, A.; Chung, C. W.; Foley, J.; Gordon, L.; Grandi, P.; Gray, J. R. J.; Harrison, L. A.; Kruger, R. G.; Matteo, J. J.; McCabe, M. T.; Messenger, C.; Mitchell, D.; Phillipou, A.; Preston, A.; Prinjha, R. K.; Rianjongdee, F.; Rioja, I.; Taylor, S.; Wall, I. D.; Watson, R. J.; Woolven, J. M.; Wyce, A.; Zhang, X. P.; Demont, E. H. Fragment-Based Scaffold Hopping: Identification of Potent, Selective, and Highly Soluble Bromo and Extra Terminal Domain (BET) Second Bromodomain (BD2) Inhibitors. *J. Med. Chem.* **2021**, *64* (15), 10772–10805.

(55) Whiting, R. L.; Choppin, A.; Luehr, G.; Jasper, J. R. Preclinical Evaluation of the Effects of Trazpiroben (TAK-906), a Novel, Potent Dopamine D2/D3 Receptor Antagonist for the Management of Gastroparesis. *J. Pharmacol. Exp. Ther.* **2021**, *379* (1), 85–95.

(56) Nirogi, R.; Mohammed, A. R.; Shinde, A. K.; Gagginapally, S. R.; Kancharla, D. M.; Ravella, S. R.; Bogaraju, N.; Middekadi, V. R.; Subramanian, R.; Palacharla, R. C.; Benade, V.; Muddana, N.; Abraham, R.; Medapati, R. B.; Thentu, J. B.; Mekala, V. R.; Petlu, S.; Lingavarapu, B. B.; Yarra, S.; Kagita, N.; Goyal, V. K.; Pandey, S. K.; Jasti, V. Discovery and Preclinical Characterization of Usmarapride (SUVN-D4010): A Potent, Selective 5-HT4 Receptor Partial Agonist for the Treatment of Cognitive Deficits Associated with Alzheimer's Disease. *J. Med. Chem.* **2021**, *64* (15), 10641–10665.

(57) Liang, J.; Zbieg, J. R.; Blake, R. A.; Chang, J. H.; Daly, S.; Dipasquale, A. G.; Friedman, L. S.; Gelzleichter, T.; Gill, M.; Giltnane, J. M.; Goodacre, S.; Guan, J.; Hartman, S. J.; Ingalla, E. R.; Kategaya, L.; Kiefer, J. R.; Kleinheinz, T.; Labadie, S. S.; Lai, T.; Li, J.; Liao, J.; Liu, Z.; Mody, V.; McLean, N.; Metcalfe, C.; Nannini, M. A.; Oeh, J.; O'Rourke, M. G.; Ortwine, D. F.; Ran, Y.; Ray, N. C.; Roussel, F.; Sambrone, A.; Sampath, D.; Schutt, L. K.; Vinogradova, M.; Wai, J.; Wang, T.; Wertz, I. E.; White, J. R.; Yeap, S. K.; Young, A.; Zhang, B.; Zheng, X.; Zhou, W.; Zhong, Y.; Wang, X. GDC-9545 (Giredestrant): A Potent and Orally Bioavailable Selective Estrogen Receptor Antagonist and Degrader with an Exceptional Preclinical Profile for ER+ Breast Cancer. *J. Med. Chem.* **2021**, *64* (16), 11841–11856.

(58) Okimoto, R.; Ino, K.; Ishizu, K.; Takamatsu, H.; Sakamoto, K.; Yuyama, H.; Fuji, H.; Someya, A.; Ohtake, A.; Ishigami, T.; Masuda, N.; Takeda, M.; Kajioka, S.; Yoshimura, N. Potentiation of Muscarinic M3 Receptor Activation through a New Allosteric Site with a Novel Positive Allosteric Modulator ASP8302. *J. Pharmacol. Exp. Ther.* **2021**, *379* (1), 64–73.

(59) Klein, M.; Busch, M.; Friese-Hamim, M.; Crosignani, S.; Fuchss, T.; Musil, D.; Rohdich, F.; Sanderson, M. P.; Seenisamy, J.; Walter-Bausch, G.; Zanelli, U.; Hewitt, P.; Esdar, C.; Schadt, O. Structure-Based Optimization and Discovery of M3258, a Specific Inhibitor of the Immunoproteasome Subunit LMP7 (B5i). *J. Med. Chem.* **2021**, *64* (14), 10230–10245.

(60) Gajewiak, J.; Christensen, S. B.; Dowell, C.; Hararah, F.; Fisher, F.; Huynh, P. N.; Olivera, B. M.; McIntosh, J. M. Selective Penicillamine Substitution Enables Development of a Potent Analgesic Peptide That Acts through a Non-Opioid-Based Mechanism. *J. Med. Chem.* **2021**, *64* (13), 9271–9278.

(61) Perry, M. W. D.; Björhall, K.; Bold, P.; Brűlls, M.; Börjesson, U.; Carlsson, J.; Chang, H. F. A.; Chen, Y.; Eriksson, A.; Fihn, B. M.; Fransson, R.; Fredlund, L.; Ge, H.; Huang, H.; Karabelas, K.; Lamm Bergström, E.; Lever, S.; Lindmark, H.; Mogemark, M.; Nikitidis, A.; Palmgren, A. P.; Pemberton, N.; Petersen, J.; Rodrigo Blomqvist, M.; Smith, R. W.; Thomas, M. J.; Ullah, V.; Tyrchan, C.; Wennberg, T.; Westin Eriksson, A.; Yang, W.; Zhao, S.; Öster, L. Discovery of

AZD8154, a Dual PI3Kγδ Inhibitor for the Treatment of Asthma. *J. Med. Chem.* **2021**, *64* (12), 8053–8075.

(62) Grand, D. Le; Gosling, M.; Baettig, U.; Bahra, P.; Bala, K.; Brocklehurst, C.; Budd, E.; Butler, R.; Cheung, A. K.; Choudhury, H.; Collingwood, S. P.; Cox, B.; Danahay, H.; Edwards, L.; Everatt, B.; Glaenzel, U.; Glotin, A. L.; Groot-Kormelink, P.; Hall, E.; Hatto, J.; Howsham, C.; Hughes, G.; King, A.; Koehler, J.; Kulkarni, S.; Lightfoot, M.; Nicholls, I.; Page, C.; Pergl-Wilson, G.; Popa, M. O.; Robinson, R.; Rowlands, D.; Sharp, T.; Spendiff, M.; Stanley, E.; Steward, O.; Taylor, R. J.; Tranter, P.; Wagner, T.; Watson, H.; Williams, G.; Wright, P.; Young, A.; Sandham, D. A. Discovery of Icenticaftor (QBW251), a Cystic Fibrosis Transmembrane Conductance Regulator Potentiator with Clinical Efficacy in Cystic Fibrosis and Chronic Obstructive Pulmonary Disease. *J. Med. Chem.* **2021**, *64* (11), 7241–7260.

(63) Barilli, A.; Aldegheri, L.; Bianchi, F.; Brault, L.; Brodbeck, D.; Castelletti, L.; Feriani, A.; Lingard, I.; Myers, R.; Nola, S.; Piccoli, L.; Pompilio, D.; Raveglia, L. F.; Salvagno, C.; Tassini, S.; Virginio, C.; Sabat, M. From High-Throughput Screening to Target Validation: Benzo[d]Isothiazoles as Potent and Selective Agonists of Human Transient Receptor Potential Cation Channel Subfamily M Member 5 Possessing In Vivo Gastrointestinal Prokinetic Activity in Rodents. *J. Med. Chem.* **2021**, *64* (9), 5931–5955.

(64) Quiroz, R. V.; Reutershan, M. H.; Schneider, S. E.; Sloman, D.; Lacey, B. M.; Swalm, B. M.; Yeung, C. S.; Gibeau, C.; Spellman, D. S.; Rankic, D. A.; Chen, D.; Witter, D.; Linn, D.; Munsell, E.; Feng, G.; Xu, H.; Hughes, J. M. E.; Lim, J.; Saurí, J.; Geddes, K.; Wan, M.; Mansueto, M. S.; Follmer, N. E.; Fier, P. S.; Siliphaivanh, P.; Daublain, P.; Palte, R. L.; Hayes, R. P.; Lee, S.; Kawamura, S.; Silverman, S.; Sanyal, S.; Henderson, T. J.; Ye, Y.; Gao, Y.; Nicholson, B.; Machacek, M. R. The Discovery of Two Novel Classes of 5,5-Bicyclic Nucleoside-Derived PRMT5 Inhibitors for the Treatment of Cancer. *J. Med. Chem.* **2021**, *64* (7), 3911–3939.

(65) Pelat, M.; Barbe, F.; Daveu, C.; Ly-Nguyen, L.; Lartigue, T.; Marque, S.; Tavares, G.; Ballet, V.; Guillon, J. M.; Steinmeyer, K.; Wirth, K.; Gögelein, H.; Arndt, P.; Rackelmann, N.; Weston, J.; Bellevergue, P.; McCort, G.; Trellu, M.; Lucats, L.; Beauverger, P.; Pruniaux-Harnist, M. P.; Janiak, P.; Chézalviel-Guilbert, F. SAR340835, a Novel Selective Na+/Ca2+ Exchanger Inhibitor, Improves Cardiac Function and Restores Sympathovagal Balance in Heart Failure. *J. Pharmacol. Exp. Ther.* **2021**, *377* (2), 293–304.

(66) Shen, Y.; Li, F.; Szewczyk, M. M.; Halabelian, L.; Chau, I.; Eram, M. S.; Dela Seña, C.; Park, K. S.; Meng, F.; Chen, H.; Zeng, H.; Dong, A.; Wu, H.; Trush, V. V.; Mcleod, D.; Zepeda-Velázquez, C. A.; Campbell, R. M.; Mader, M. M.; Watson, B. M.; Schapira, M.; Arrowsmith, C. H.; Al-Awar,

R.; Barsyte-Lovejoy, D.; Kaniskan, H. Ü.; Brown, P. J.; Vedadi, M.; Jin, J. A First-in-Class, Highly Selective and Cell-Active Allosteric Inhibitor of Protein Arginine Methyltransferase 6. *J. Med. Chem.* **2021**, *64* (7), 3697–3706.

(67) Lin, H.; Sharabi, K.; Lin, L.; Ruiz, C.; Zhu, D.; Cameron, M. D.; Novick, S. J.; Griffin, P. R.; Puigserver, P.; Kamenecka, T. M. Structure-Activity Relationship and Biological Investigation of SR18292 (16), a Suppressor of Glucagon-Induced Glucose Production. *J. Med. Chem.* **2021**, *64* (2), 980–990.

(68) Peng, Y.; Zhang, Q.; Welsh, W. J. Novel Sigma 1 Receptor Antagonists as Potential Therapeutics for Pain Management. *J. Med. Chem.* **2021**, *64* (1), 890–904.

(69) Gou, X.; Yu, X.; Bai, D.; Tan, B.; Cao, P.; Qian, M.; Zheng, X.; Chen, L.; Shi, Z.; Li, Y.; Ye, F.; Liang, Y.; Ni, J. Pharmacology and Mechanism of Action of HSK16149, a Selective Ligand of α 2 δ Subunit of Voltage-Gated Calcium Channel with Analgesic Activity in Animal Models of Chronic Pain. *J. Pharmacol. Exp. Ther.* **2021**, *376* (3), 330–337.

(70) Hino, N.; Marumo, T.; Kotani, M.; Shimazaki, T.; Kaku-Fukumoto, A.; Hikichi, H.; Karasawa, J. I.; Tomishima, Y.; Komiyama, H.; Tatsuda, E.; Nozawa, D.; Nakamura, T.; Chaki, S. A Novel Potent and Selective Histamine H3 Receptor Antagonist Enerisant: In Vitro Profiles, In Vivo Receptor Occupancy, and Wake-Promoting and Procognitive Effects in Rodents. *J. Pharmacol. Exp. Ther.* **2020**, *375* (2), 276–285.

(71) Hjorth, S.; Waters, S.; Waters, N.; Tedroff, J.; Svensson, P.; Fagerberg, A.; Edling, M.; Svanberg, B.; Ljung, E.; Gunnergren, J.; McLean, S. L.; Grayson, B.; Idris, N. F.; Neill, J. C.; Sonesson, C. (3 S)-3-(2,3-Difluorophenyl)-3-Methoxypyrrolidine (IRL752) -a Novel Cortical-Preferring Catecholamine Transmission- and Cognition-Promoting Agent. *J. Pharmacol. Exp. Ther.* **2020**, *374* (3), 404–419.

(72) Guillot, E.; Le Bail, J. C.; Paul, P.; Fourgous, V.; Briand, P.; Partiseti, M.; Cornet, B.; Janiak, P.; Philippo, C. Lysophosphatidic Acid Receptor Agonism: Discovery of Potent Nonlipid Benzofuran Ethanolamine Structures. *J. Pharmacol. Exp. Ther.* **2020**, *374* (2), 283–294.

(73) Waters, S.; Sonesson, C.; Svensson, P.; Tedroff, J.; Carta, M.; Ljung, E.; Gunnergren, J.; Edling, M.; Svanberg, B.; Fagerberg, A.; Kullingsjö, J.; Hjorth, S.; Waters, N. Preclinical Pharmacology of [2-(3-Fluoro-5-Methanesulfonyl-Phenoxy)Ethyl](Propyl)Amine (IRL790), a Novel Dopamine Transmission Modulator for the Treatment of Motor and Psychiatric Complications in Parkinson Disease. *J. Pharmacol. Exp. Ther.* **2020**, *374* (1), 113–125.

(74) Ozoux, M. L.; Briand, V.; Pelat, M.; Barbe, F.; Schaeffer, P.; Beauverger, P.; Poirier, B.; Guillon, J. M.; Petit, F.; Altenburger, J. M.; Bidouard, J. P.; Janiak, P. Potential Therapeutic Value of Urotensin II Receptor Antagonist in Chronic Kidney Disease and Associated Comorbidities. *J. Pharmacol. Exp. Ther.* **2020**, *374* (1), 24–37.

(75) Roberts, A.; Grafton, G.; Powell, A. D.; Brock, K.; Chen, C.; Xie, D.; Huang, J.; Liu, S.; Cooper, A. J.; Brady, C. A.; Qureshi, O.; Stamataki, Z.; Manning, D. D.; Moore, N. A.; Sargent, B. J.; Guzzo, P. R.; Barnes, N. M. CSTI-300 (SMP-100); a Novel 5-HT3 Receptor Partial Agonist with Potential to Treat Patients with Irritable Bowel Syndrome or Carcinoid Syndrome. *J. Pharmacol. Exp. Ther.* **2020**, *373* (1), 122–134.

(76) Hargrove, D. M.; Alagarsamy, S.; Croston, G.; Laporte, R.; Qi, S.; Srinivasan, K.; Sueiras-Diaz, J.; Wisniewski, K.; Hartwig, J.; Lu, M.; Posch, A. P.; Wisniewska, H.; Schteingart, C. D.; Rivière, P. J. M.; Dimitriadou, V. Pharmacological Characterization of Apraglutide, a Novel Long-Acting Peptidic Glucagon-Like Peptide-2 Agonist, for the Treatment of Short Bowel Syndrome. *J. Pharmacol. Exp. Ther.* **2020**, *373* (2), 193–203.

(77) Moore, E.; Fraley, M. E.; Bell, I. M.; Burgey, C. S.; White, R. B.; Li, C. C.; Regan, C. P.; Danziger, A.; Michener, M. S.; Hostetler, E.; Banerjee, P.; Salvatore, C. Characterization of Ubrogepant: A Potent and Selective Antagonist of the Human Calcitonin Gene–Related Peptide Receptor. *J. Pharmacol. Exp. Ther.* **2020**, *373* (1), 160–166.

(78) Wyatt, R. M.; Fraser, I.; Welty, N.; Lord, B.; Wennerholm, M.; Sutton, S.; Ameriks, M. K.; Dugovic, C.; Yun, S.; White, A.; Nguyen, L.; Koudriakova, T.; Tian, G.; Suarez, J.; Szewczuk, L.; Bonnette, W.; Ahn, K.; Ghosh, B.; Flores, C. M.; Connolly, P. J.; Zhu, B.; Macielag, M. J.; Brandt, M. R.; Chevalier, K.; Zhang, S. P.; Lovenberg, T.; Bonaventure, P. Pharmacologic Characterization of JNJ-42226314, [1-(4-Fluorophenyl)Indol-5-Yl]-[3-[4-(Thiazole-2-Carbonyl)Piperazin-1-Yl]Azetidin-1-Yl]Methanone, a Reversible, Selective, and Potent Monoacylglycerol Lipase Inhibitor. *J. Pharmacol. Exp. Ther.* **2020**, *372* (3), 339–353.

(79) Maguire, D. R.; Gerak, L. R.; Cami-Kobeci, G.; Husbands, S. M.; France, C. P.; Belli, B.; Flynn, P. OREX-1019: A Novel Treatment of Opioid Use Disorder and Relapse Prevention. *J. Pharmacol. Exp. Ther.* **2020**, *372* (2), 205–215.

(80) Wood, M.; Daniels, V.; Provins, L.; Wolff, C.; Kaminski, R. M.; Gillard, M. Pharmacological Profile of the Novel Antiepileptic Drug Candidate Padsevonil: Interactions with Synaptic Vesicle 2 Proteins and the GABAA Receptor. *J. Pharmacol. Exp. Ther.* **2020**, *372* (1), 1–10.

(81) Rosenbrock, H.; Giovannini, R.; Schänzle, G.; Koros, E.; Runge, F.; Fuchs, H.; Marti, A.; Reymann, K. G.; Schröder, U. H.; Fedele, E.; Dorner-Ciossek, C. The Novel Phosphodiesterase 9A Inhibitor BI 409306 Increases Cyclic Guanosine Monophosphate Levels in the Brain, Promotes Synaptic Plasticity, and Enhances Memory Function in Rodents. *J. Pharmacol. Exp. Ther.* **2019**, *371* (3), 633–641.

(82) Enomoto, T.; Tatara, A.; Goda, M.; Nishizato, Y.; Nishigori, K.; Kitamura, A.; Kamada, M.; Taga, S.; Hashimoto, T.; Ikeda, K.; Fujii, Y. A Novel Phosphodiesterase 1 Inhibitor DSR-141562 Exhibits Efficacies in Animal Models for Positive, Negative, and Cognitive Symptoms Associated with Schizophrenia. *J. Pharmacol. Exp. Ther.* **2019**, *371* (3), 692–702.

(83) Takagahara, S.; Shinohara, H.; Itokawa, S.; Satomi, Y.; Ando, A.; Yamamoto, T.; Suzuki, H.; Fujimoto, T.; Kubo, K.; Ikeda, S. A Novel Orally Available Delta-5 Desaturase Inhibitor Prevents Atherosclerotic Lesions Accompanied by Changes in Fatty Acid Composition and Eicosanoid Production in ApoE Knockout Mice. *J. Pharmacol. Exp. Ther.* **2019**, *371* (2), 290–298.

(84) Dedic, N.; Jones, P. G.; Hopkins, S. C.; Lew, R.; Shao, L.; Campbell, J. E.; Spear, K. L.; Large, T. H.; Campbell, U. C.; Hanania, T.; Leahy, E.; Koblan, K. S. SEP-363856, a Novel Psychotropic Agent with a Unique, Non-D2 Receptor Mechanism of Action. *J. Pharmacol. Exp. Ther.* **2019**, *371* (1), 1–14.

(85) Aparici, M.; Carcasona, C.; Ramos, I.; Montero, J. L.; Otal, R.; Ortiz, J. L.; Cortijo, J.; Puig, C.; Vilella, D.; De Alba, J.; Doe, C.; Gavaldà, A.; Miralpeix, M. Pharmacological Profile of AZD8871 (LAS191351), a Novel Inhaled Dual M3 Receptor Antagonist/ β 2-Adrenoceptor Agonist Molecule with Long-Lasting Effects and Favorable Safety Profile. *J. Pharmacol. Exp. Ther.* **2019**, *370* (1), 127–136.
